# A review on the cultivation, bioactive compounds, health-promoting factors and clinical trials of medicinal mushrooms *Taiwanofungus camphoratus*, *Inonotus obliquus* and *Tropicoporus linteus*

**DOI:** 10.1186/s40694-024-00176-3

**Published:** 2024-07-10

**Authors:** Phoebe Yon Ern Tee, Thiiben Krishnan, Xin Tian Cheong, Snechaa A. P. Maniam, Chung Yeng Looi, Yin Yin Ooi, Caroline Lin Lin Chua, Shin-Yee Fung, Adeline Yoke Yin Chia

**Affiliations:** 1 School of Biosciences, Faculty of Health and Medical Sciences, Taylor’s University, 47500 Selangor, Malaysia; 2 Department of Molecular Medicine, Faculty of Medicine Building, University of Malaya, 50603 Kuala Lumpur, Malaysia

**Keywords:** Bioactive compounds, Health-promoting, Medicinal mushroom, Natural product, Nutraceuticals, Therapeutic properties

## Abstract

Medicinal mushrooms, such as *Taiwanofungus camphoratus*, *Inonotus obliquus*, and *Tropicoporus linteus*, have been used in traditional medicine for therapeutic purposes and promotion of overall health in China and many East Asian countries for centuries. Modern pharmacological studies have demonstrated the large amounts of bioactive constituents (such as polysaccharides, triterpenoids, and phenolic compounds) available in these medicinal mushrooms and their potential therapeutic properties. Due to the rising demand for the health-promoting medicinal mushrooms, various cultivation methods have been explored to combat over-harvesting of the fungi. Evidence of the robust pharmacological properties, including their anticancer, hypoglycemic, hypolipidemic, antioxidant, and antiviral activities, have been provided in various studies, where the health-benefiting properties of the medicinal fungi have been further proven through numerous clinical trials. In this review, the cultivation methods, available bioactive constituents, therapeutic properties, and potential uses of *T. camphoratus*, *I. obliquus* and *T. linteus* are explored.

## Introduction

Natural products, consisting of an extensive range of structural and chemical diversity, have been used as therapeutic agents for various human ailments and diseases for many years [1]. Natural products and their derivatives have been approved for use in various fields, including oncology, as well as in the anti-infective area [2]. The biological activities of these natural sources can be attributed to the presence of medicinal compounds found in them, such as polysaccharides and terpenoids [3].

Medicinal mushrooms, including *Taiwanofungus camphoratus*, *Inonotus obliquus*, and *Tropicoporus linteus*, are rich sources of these bioactive constituents. Hispidin, ergosterol peroxide, protocatechuic acid, and betulin are all biologically-active compounds that can be found in the medicinal mushrooms. Having a long history of use in traditional and folk medicine, they have been used for the amelioration of conditions ranging from cancer [4] to heart and liver disease [5]. Modern scientific research has validated their claimed properties, where the increasingly developed field of medical mushroom research demonstrated the potent activities of bioactive compounds isolated from the fungi. They contain great potential to treat a wide range of conditions, including tumor [6, 7], diabetes [8–10], viral infections [11–13], and disease related to oxidative stress [14–16]. Evidence of their health-benefiting properties have been established through various studies and clinical trials [17]. Therefore, due to their significant pharmacological and health-promoting properties, various efforts have been made to increase their production sustainably [18].

The market value of medicinal mushrooms and their derived products was estimated to be USD 6.0 billion in 1999, with demand increasing between 20 and 40% annually since then [19]. With a selling price of up to US$25,000/kg, the annual output value of *T. camphoratus* in Taiwan is estimated to be about NT$3 billion [20] and a global market value of $100 million per year [21]. For *I. obliquus*, the current global market value of US$28.2 billion is expected to rise to US$87 billion by 2034 [22], while total sales of various *T. linteus* products in Japan and South Korea alone in 2023 have exceeded 10 billion yen (US$65 million) [23].

In view of the rising demand and expanding market potential, this review aims to explore the growth and cultivation, bioactive components, therapeutic potentials, medical evidence of the pharmacological properties of medicinal mushrooms *Taiwanofungus camphoratus*, *Inonotus obliquus*, and *Tropicoporus linteus*, with a goal of providing a useful reference for necessary information for the further study of the fungi.

## Growth and cultivation

### Taiwanofungus camphoratus

**Fig. 1:**
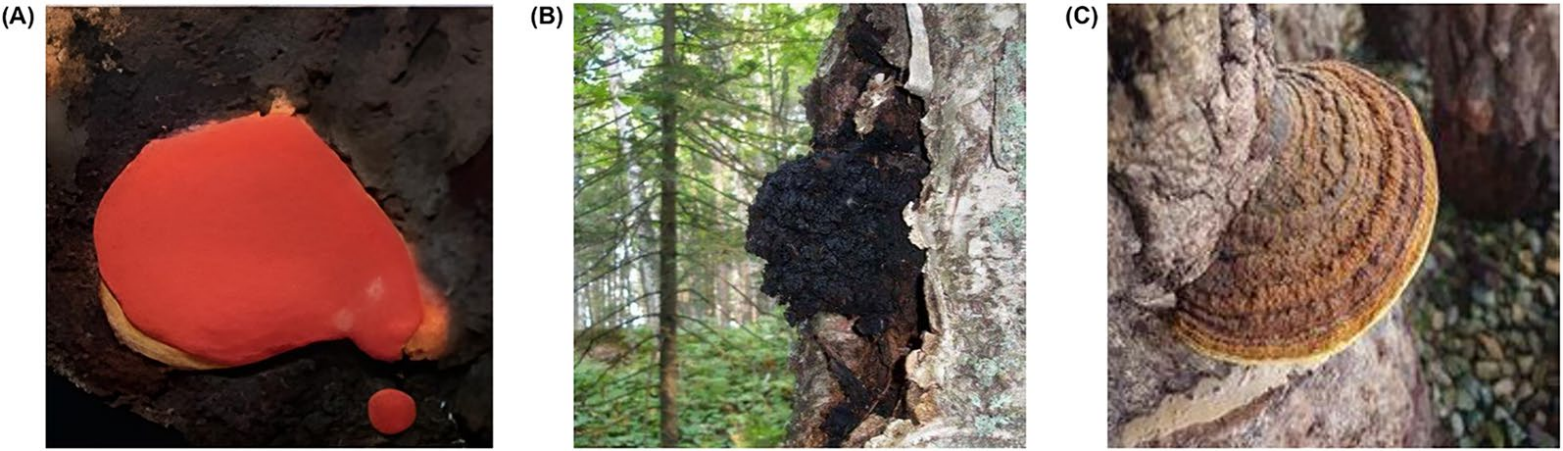
Fruiting bodies of (**A**) *Taiwanofungus camphoratus*, (**B**) *Inonotus obliquus*, and (**C**) *Tropicoporus linteus* on their host trees [25, 26] (Min and Kang 2021)

*T. camphoratus* thrives in the natural habitat of Taiwan's mountainous regions and grows at altitudes between 450 and 2000 m, where the *Cinnamomum kanehirai* tree serves as its primary nutrient source [24]. A distinguishing feature of *T. camphoratus* is its growth pattern, whereby the fruiting body can only develop to full maturity when the host tree has naturally aged away (Fig. 1).

In the past, unsustainable methods have been practiced due to the high demand for wild *T. camphoratus* and the widespread use of *C. kanehirai* wood for furniture production. Fuelled by the need to satisfy this demand, excessive harvesting of the mushroom and its host tree has led to disastrous consequences on the availability of the fungi and its habitat [27]. Realizing the ecological importance of *C. kanehirai* and *T. camphoratus*’ reliance on this species, the Taiwanese government has intervened by setting conservation measures in place to prevent overexploitation and maintain the ecosystem’s ideal state [28]. However, due to its limited availability and difficult cultivation, *T. camphoratus* remains expensive and is often referred to as the “ruby in Taiwanese forests” [29].

Although between 80 and 85% of medicinal mushroom products are obtained from fruiting bodies, the continual rise in market demand has made mycelial products the wave of the future [30]. Artificial cultivation methods for *T. camphoratus* were established in order to reduce the effects of overexploitation and sustainably satisfy market demand. Two main cultivation techniques have been commonly utilized to meet the increasing demand, namely submerged fermentation and solid-state fermentation [31]. Each method possesses its own advantages and limitations, which have been described in Table 1. For example, the ability to precisely control physical, chemical, and biological factors using submerged fermentation allows high reproducibility of metabolites, thus increasing the ease of scale-up for industrial production purposes [32, 33]. Furthermore, recent efforts have been made to enhance the efficiencies of cultivation methods, such as the application of low-frequency alternating magnetic field to enhance mycelium growth, expression of genes involved in amino acid metabolism and synthesis, and accumulation of active metabolites in cultivated *T. camphoratus* [18].

**Table 1:** A comparison between two widely used techniques for the cultivation of medicinal mushrooms [34–38]

Cultivation techniques	Advantages	Disadvantages
Submerged fermentation	Shorter fermentation period Precise control of growth parameters Ease of monitoring growth Culture homogeneity by agitation High reproducibility Feasible for production on industrial scale	Expensive equipment (bioreactors) required Require skilled operators for operation and maintenance of bioreactors Product dilution in liquid medium The increase in broth viscosity can: - Create obstacles for even distribution of oxygen and removal of carbon dioxide - Create function disorders of bioreactors
Solid-state fermentation	Allows use of agro-industrial waste as substrate Low power consumption Lower cost Better oxygen circulation Simulates natural environment of mushroom growth Undiluted product at the end of fermentation	High variability of growth parameters Low reproducibility Not suitable for production on industrial scale Inhomogeneous mixing of culture nutrients Higher impurity product

### Inonotus obliquus

The Chaga mushroom, scientifically known as *I. obliquus,* is a parasitic fungus belonging to the family of *Hymenochaetaceae* that gained attention for its unique appearance and potential health benefits [39]. The development of *I. obliquus* can be recognized by a canker-like look in a shade of dark brown to black, resembling burnt wood, which is a result of fungal mycelium accumulation [40]. Despite its slow growth, *I. obliquus* ultimately reaches maturity, developing a distinctive texture and occasionally exposing its inner yellow flesh. The fungi frequently cohabit with birch trees in moist, wetland habitats through symbiotic interactions, whereby regions such as Russia, Korea, China, Eastern and Northern Europe, northern parts of the United States, and portions of Canada are included in their geographic distribution [41].

*I. obliquus* must be harvested using special procedures to protect the wellbeing of both the host tree and the fungi. It can be carefully removed from a tree's bark using equipments such as hatchets or hammers without harming the inner layers [42]. The optimal time to harvest *I. obliquus* is in late winter when the host tree is dormant and nutrient accumulation is at its greatest. This method allows for repeated regeneration of *I. obliquus* on the same tree while protecting the health of the host tree. A complex relationship exists between *I. obliquus* and its host tree, whereby the viability of the fungi declines with the health of the tree [42]. Thus, it is essential to understand the symbiotic relationship between *I. obliquus* and its host to enable appropriate cultivation methods for this priceless natural resource.

Due to the slow-growing nature and hence limited supply of *I. obliquus*, various cultivation methods have been employed to address this issue. A 2005 study on the optimum culture condition of *I. obliquus* provided valuable basic information on the in vitro cultivation conditions, including the media used and additions such as amino acids and yeast extract for the improvement of fungal growth and biomass production [43]. Submerged fermentation has been commonly used for the production of *I. obliquus*, whereby careful optimisation of optimum broth formula for submerged culturing of *I. obliquus* mycelium has successfully enhanced mycelial biomass yields [44]. Other efforts have also been made to determine the optimum *I. obliquus* strains and cultivation substrates for producing productive strains of the medicinal fungi, where the use of *Betula* wood as inoculation dowels was suggested to shorten production time [45].

### Tropicoporus linteus

*T. linteus* is a well-known medicinal fungus that has drawn the interest of researchers and traditional medicine practitioners. It is known by several names, including "sanghuang" in China, "meshimakobu" in Japan, and "sangwhang" in Korea. Due to limited availability in the wild, its development and harvest present added challenges.

*T. linteus* is usually found on the trunks *Populus* Linn., *Quercus* Linn., *Toxicodendron vernicifluum* and *Morus alba* Linn. in its natural habitat [46]. It stands out in the fungi world due to its unusual pileate, perennial, horseshoe-shaped basidiocarps [46]. When dried, the pore surface changes from rusty brown to brown, the pial surface from a dark brown hue to black, and the tubes attain a cinnamon-yellowish-brown tint. The months of April and May are when *T. linteus* is most suitable for harvesting in the wild due to favourable environmental circumstances for its growth [47].

Despite its significant therapeutic value, the availability of *T. linteus* for traditional medicine and industrial use has been constrained by its low abundance in its natural environment, where the demand for this rare resource simply exceeds what the wild population can supply [48]. Researchers and cultivators have concentrated on artificially producing *T. linteus* to address this issue, where cultivation procedures to produce fruiting bodies often require growing it on solid artificial substrate [49]. Optimal submerged culture composition and conditions for maximum mycelial biomass have also been determined, enabling a more sustainable and steady supply for medical and scientific uses, and utilization of this unique fungus to its maximum potential [50]. Furthermore, artificial cultivation of *T. linteus* strains on oak logs has been successful, with resulting polysaccharides retaining their valuable bioactive properties, including immunomodulatory and anticancer effects [51].

## Bioactive compounds

**Fig. 2:**
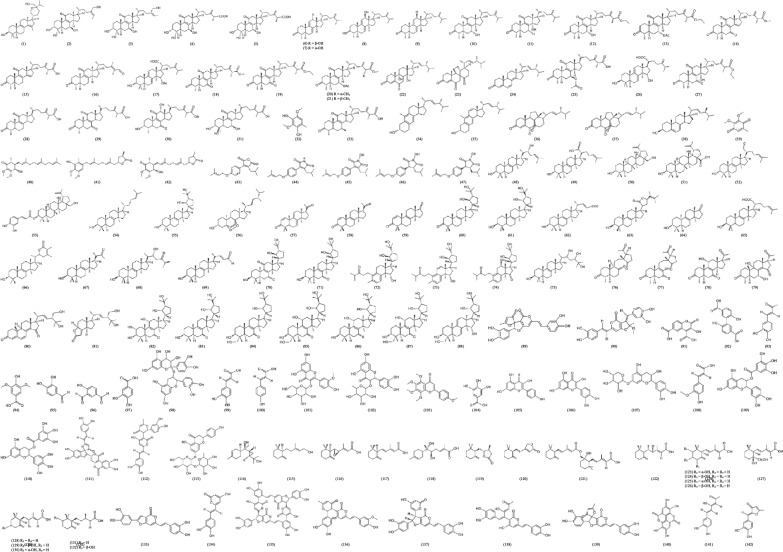
Chemical structures of bioactive compounds isolated from *Taiwanofungus camphoratus* (1–38 terpenes and terpenoids; 39–42 quinone derivatives; 43–47 maleic and succinic acid derivatives), *Inonotus obliquus* (38, 48–88 terpenes and terpenoids; 89–113 phenolic compounds), and *Tropicoporus linteus* (114–132 terpenes and terpenoids; 93, 95, 97, 99, 111, 112, 133–142 phenolic compounds)

The chemical structures of bioactive compounds isolated from the three medicinal mushrooms explored in this review are presented in (Fig. 2).

### Taiwanofungus camphoratus

#### Polysaccharides

A water-soluble polysaccharide, composed of β-D-glucan, was isolated and purified from cultured *Taiwanofungus camphoratus* powder [52]. The polysaccharide was found to have a molecular weight of 17.2 kDa, where further structural elucidation revealed a segmental and repetitive structure of the molecule, composed of 1,3,6-β-d-glucose, 1,3-β-d-glucose, and 1,4-α-d-galactose at a 2.21:1.00 mol ratio of glucose to galactose. The β-d-glucan was demonstrated to exert anti-inflammatory and anti-oxidation activities in lipopolysaccharide (LPS) induced-human hepatocytes to thus provide a hepatoprotective effect. An exopolysaccharide [53] and galactoglucan primarily composed of galactose (1,6-β-d-galactose) and glucose (1,6-β-d-glucose, 1,3-α-d-glucose, and 1,4-β-d-glucose), with terminal α-d-6-deoxyglucose and α-d-glucose [54] isolated from *T. camphoratus* also exhibited inflammation-reducing capabilities.

Three other polysaccharides from the cell wall of *T. camphoratus* mycelia obtained through hot water, cold alkali, and hot alkali extraction were demonstrated to also mainly consist of glucose, with molar percentages of 75.32, 78.06, and 87.28 respectively [55]. The hot water extracted polysaccharide mainly consisted of 1,4-d-glucose, while those isolated through cold and hot alkali extractions were primarily composed of 1,3-d-glucose and were reported to contain potent in vitro antioxidant properties, suggesting the potential contribution of 1,3-d-glucose towards the observed activities exerted by the alkali-soluble polysaccharides. Another polysaccharide extracted from the mycelia of *T. camphoratus* was shown to possess antitumor activities [56]. Contrary to the polysaccharides isolated by previous researchers [52, 53, 55], this polysaccharide only contained trace amounts of glucose, as it is mainly composed of mannose, xylose, arabinose, fucose, and rhamnose in a ratio of 31.27:1.77:1.44:1.34:1.00, with a backbone composed of repeating α-1,3-, α-1,6-, α-1,2-, and α-1,4-glycosidic linkages.

Recently, sulfated polysaccharides obtained from *T. camphoratus* grown in zinc sulfate enriched culture conditions were studied by Lee et al. [57]. It was found that the sulfated polysaccharides not only contain inflammation-inhibiting properties but also possess anti-lung cancer activities. In a comparison of sulfated polysaccharides to non-sulfated groups, sulfated derivatives showed more robust anti-inflammatory effects than non-sulfated polysaccharides, thus suggesting the role of sulfation in the inflammation-reducing capacities of the bioactive compound [58]. In another study, a 13.5 kDa heterogalactan was extracted from *T. camphoratus* and found to be composed of α-d-1,6-linked galactose backbone chain with terminal α-d-mannose and α-l-fucose every six galactose residues [59]. Similar to the results observed by Chen et al. [58], the mannofucogalactan itself did not demonstrate inhibitory effects on angiogenesis, while its sulfated derivative exerted a significant dose-dependent reduction of tube formation.

Besides sulfated derivatives, selenium-enriched polysaccharides have also been analyzed [39]. It was reported that in vitro scavenging efficiency was greatly enhanced following selenium enrichment, thus making selenium-enriched *T. camphoratus* polysaccharides a promising antioxidant agent for food and pharmaceutical applications.

It is noteworthy to mention that besides from the variety of bioactive components, alkali-extracted dietary fiber from the residues of *T. camphoratus* basswood cultured fruiting body also serves as a promising source of functional ingredients [60]. The study highlighted that the dietary fiber promoted RAW 264.7 cell proliferation, phagocytosis, nitric oxide (NO) production, and release of pro-inflammatory cytokines tumour necrosis factor alpha (TNF-α) and interleukin-1 beta (IL-1β), thus revealing its immunomodulatory properties, and possibly other bioactivities.

### Terpenes and terpenoids

Evaluation of new ergostane-type triterpenoids, antcamphorols A-K, (**1–11**) revealed ROS scavenging abilities of antcamphorols G (**7**), I (**9**), and J (**10**), along with cytotoxic activities of antcamphorols C and H against U251 and MCF-7 [61]. Furthermore, through isolation from dish cultures of *T. camphoratus*, three new (**12–14**) and 10 known isolated triterpenoids (**15–24**) were demonstrated to possess cytotoxic activities on cancer cell lines HL60, U251, SW480, and MCF-7 [62]. Interestingly, it was found that the combination of two of the isolated triterpenoids (**25, 26**) could enhance the cytotoxic effect of paclitaxel, an established anti-cancer agent, suggesting triterpenoids as potential sensitizers of paclitaxel for chemotherapy. Antcamphin Z (**27**) also displayed potent cytotoxic activities against human cancer cells (U251, HL60, SW480, and A549) with no significant effects on normal cells (LO2) [63]. Besides their cytotoxic activities, triterpenoids antcins A (**28**) [64], B (**29**), H (**30**), and K (**31**) [65] have also been reported to exert an anti-inflammatory effect.

Using chiral-supercritical fluid chromatography (SFC), R/S epimeric pairs of ergostane triterpenoids, including antcin A (**28**) and antcin B (**29**), were separated from *T. camphoratus* at a higher efficiency than high-performance liquid chromatography (HPLC) [66]. This thus demonstrates chiral-SFC as a more advantageous method for separation of epimeric bioactive compounds. Mechanochemical-assisted extraction has also been used for the isolation of triterpenoids from the medicinal fungus, where extracts containing triterpenoids (**32**, **33**) obtained through this technique led to more robust antioxidant, anti-inflammatory, and immunomodulatory activities than ethanol thermal reflux-extracted compounds [67].

Steroids (**34–37**) isolated from the medicinal mushroom have exhibited cytotoxic activities against murine colorectal and human leukemia cancer cell lines [68]. Furthermore, ergosterol (**38**) obtained from *T. camphoratus* was recently found to significantly reduce nuclear factor kappa B (NF-κB) phosphorylation, microglial activation–associated ionized calcium-binding adapter molecule-1 (IBA-1), and LPS-induced neuron damage, therefore making it an effective anti-neuroinflammatory agent [69].

#### Quinone derivatives

A novel quinone derivative, coenzyme Q0 (CoQ0, 2,3-dimethoxy-5-methyl-1,4-benzoquinone) (**39**), was isolated from fermented culture broth of *T. camphoratus* by Yang et al. [70]. This new compound was found to be capable of inducing apoptosis and autophagic cell death both in vitro in human glioblastoma cells and in vivo in glioblastoma-xenografted mice, through increased reactive oxygen species (ROS) production.

Another bioactive constituent found in the mycelium of *T. camphoratus* is antroquinonol (**40**), which is a ubiquinone derivative. Antroquinonol has been reported to inhibit the migration and invasion of breast cancer cells, through suppression of matrix metalloproteinase-9 (MMP-9) and epithelial-mesenchymal transition (EMT) gene expressions [71], while antroquinonol Y (**41**) also exerts significant cytotoxic effects on human cancer cells (U251, HL60, SW480, and A549) without affecting normal cells (LO2) [63]. Furthermore, bioassay-guided isolation from methanolic extracts of *T. camphoratus* revealed a potent anti-inflammatory compound, 4-acetylantroquinonol B (**42**), which significantly inhibited polyinosinic-polycytidylic acid-induced NO production by RAW264.7 macrophages [72]. Hence, due to the highly potent anti-tumor properties and demonstrated bioactivities of antroquinonol, many studies have been conducted to enhance its synthesis [73–78].

### Maleic and succinic acid derivatives

Antrodins (**43–47**) are maleimide derivatives found in *T. camphoratus* mycelium and have been found to inhibit Hepatitis C virus (HCV) protease [79]. Antrodin A (**43**) has been reported to possess hepatoprotective properties and alleviate intestinal flora dysbiosis [80], while antrodin B (**44**) contains anti-hepatofibrotic activity [81]. Antrodin C (**45**) has been found to reduce the pathology of Alzheimer’s disease [82], protect against liver fibrosis [83], and exert cytotoxic activities against colorectal cancer cells [84].

### Inonotus obliquus

#### Polysaccharides

Polysaccharides components of *Inonotus obliquus* have been studied by many researchers for their variety of biological activities. Two polysaccharides (HIOP1-S and HIOP2-S) were obtained from *I. obliquus*, where HIOP1-S was primarily composed of glucose (29.673%) and galactose (20.547%) and HIOP2-S mainly consisted of glucose (49.881%) [85]. Interestingly, HIOP1-S consisted of both α-and β-glycosidic bonds, while HIOP2-S only contained β-type linkages. Despite the considerable divergences between the two polysaccharides, both were capable of inhibiting α-glucosidase activity and enhancing extracellular glucose consumption in insulin-resistant cells. Another study also isolated two polysaccharides from the medicinal mushroom, IOEP1 and IOEP2, that contained both α-and β-glycosidic bonds [86]. The pyran-type polysaccharides were reported to mainly consist of galactose and mannose (IOEP1) and arabinose (IOEP2), and were both also demonstrated to increase glucose consumption of insulin-resistant cells, thus exerting a hypoglycemic effect.

A polysaccharide, composed of galactose, glucose, xylose, and mannose (2.0:3.5:1.0:1.5 molar ratio), was extracted from *I. obliquus* and found to exert free-radical scavenging activities, antioxidant effects, and enhancement of nitric oxide release [87]. A polysaccharide extracted by Hu et al. [88] also exhibited antioxidant properties, and consisted of mannose, rhamnose, glucose, galactose, xylose, and arabinose in a molar ratio of 9.81:3.6:29.1:20.5:21.6:5.4. The antioxidative properties of another *I. obliquus* polysaccharide with a molecular weight of 111.9 kDa, through the regulation of the nuclear factor erythroid 2-related factor 2 (Nrf2) pathway, were found to contribute a protective effect against Alzheimer’s disease [89].

Neutral (60–73 kDa), acidic (10–31 kDa) and alkaline (> 450 kDa) polysaccharide fractions isolated from *I. obliquus* extracts, consisting primarily of 1,3- and 1,6-linked-β-glucose, were reported to possess immune-promoting activities [90]. Chen et al. [91] successfully obtained a novel polysaccharide from *I. obliquus* (IP3a). It was found that IP3a is composed of arabinose, galactose, rhamnose, and glucose (4.6:2.6:2.5:1.0), and possesses pro-apoptotic and immune-enhancing effects. A pro-apoptotic effect was also observed in a polysaccharide containing mainly glucose (74.95%), where it was found that the apoptosis-inducing effects were mediated by activation of adenosine monophosphate-activated protein kinase (AMPK) and reduction of mitochondrial membrane potential (MMP) [92].

Recently, another polysaccharide was isolated and found to have a molecular weight of 373 kDa, with a composition of 9 monosaccharides, primarily contributed by glucose (29.85%), galactose (20.70%), and mannose (14.19%) [93]. The polysaccharide was found to be capable of increasing *Firmicutes*, decreasing *Bacteroidetes*, and repairing the intestinal barrier to contribute to the protection against Type 2 diabetes mellitus. Interestingly, contrary to the findings of Su et al. [93], a polysaccharide consisting of mannose, galactose, and glucose (7.7:23.3:32.6) reduced the abundance of *Firmicutes* and enhanced *Bacteroidetes* [94], thus suggesting the impact of polysaccharide composition on biological activities.

#### Terpenes and terpenoids

Inotodiol (**48**) and trametenolic acid (**49**) are common triterpenoids found in *I. obliquus*, and were found to exert anti-proliferative activities on AGS, MCF-7, and PC3 cancer cells [95], and alleviate liver diseases through modification of the farnesoid X receptor (FXR)/small heterodimer partner (SHP)/ sterol-regulatory element binding protein-1C (SREBP-1C) axis [96]. Besides that, betulin (**50**) and betulinic acid (**51**) also inhibited the proliferation of human adenocarcinoma and human lung carcinoma cell lines, while inotodiol (**48**), 3β-hydroxy-8,24-dien-21-al (**52**), and betulin-3-*O-*caffeate (**53**) possessed immunomodulatory capabilities [97]. In addition, Zou et al. [98] highlighted the neuroprotective effects of triterpenoids lanosterol (**54**), inotodiol (**48**), and inonotsutriol A (**55**), while Park et al. [99] demonstrated the NO-inhibiting effects of inotodiol (**48**), inonotsutriol A (**55**), and trametenolic acid (**49**).

Another bioactive compound, known as ergosterol peroxide (**56**), was found to exert anti-cancer properties against colorectal cancer [100], prostatic carcinoma and breast carcinoma cells [101]. Ergosterol peroxide (**56**), ergosterol (**38**), and trametenolic acid (**49**) have also been demonstrated to have anti-inflammatory capabilities [101]. Moreover, a wide variety of *I. obliquus*-obtained triterpenoids (**57–66**) were observed to have inhibitory effects on α-glucosidase [102].

Besides known compounds, new triterpenes and triterpenoids have been elucidated and found to also contain biological activities. Two new triterpenes, inonotusane D (**67**) and inonotusol G (**68**), isolated by Zhao et al. [103] and Liu et al. [104] respectively, exhibited cytotoxic effects against cancer cells. Besides that, another new lanostane-type triterpene, inonotusane C (**69**), also exhibited cytotoxic activities against A549 and HeLa cell lines [105] thus demonstrating their chemotherapeutic potentials.

A total of 12 previously uncharacterized lanostane triterpenoids (**70–81**) were isolated by Zou et al. [98, 106], where it was revealed that compound **77** is capable of exerting neuroprotective activities. Another study also obtained novel lanostane-type triterpenoids, inonotusols H-N (**82–88**), that were found to significantly inhibit NO release by microglial cells, where inonotusols I (**83**) and L (**86**) were capable of interacting with inducible-nitric oxide synthase (iNOS) protein to hence exert a neuroprotective effect [107].

### Phenolic compounds

Phenolic compounds, including phenolic acids and flavonoids, are produced by the sclerotia of *I. obliquus* and have been found to possess potent bioactivities. A phenolic compound isolated from *I. obliquus*, identified as inoscavin A (**89**), was demonstrated to improve cell viabilities of H_2_O_2_)-injured human neuroblastoma cells to exert a neuroprotective effect through their antioxidant activities [108]. Phenolic (**91–97**) and polyphenol compounds (**98–107**), isolated by Hwang et al. [109] and Wang et al. [110] respectively were reported to contain radical scavenging activities in a dose-dependent pattern.

Melanins are another type of phenolic compound found in *I. obliquus* and have been associated with immunomodulatory properties. A water-soluble melanin fraction was found to be capable of inhibiting the complement cascade and suppressing NO production by murine macrophages [97]. Melanins isolated from aqueous extracts of *I. obliquus* also exhibited antioxidant activities, thus creating an optimum growth condition for obligate anaerobe *Bifidobacterium bifidum* [111].

A recent study revealed that ultraviolet (UV) radiation led to an increased accumulation of both extracellular and intracellular polyphenols and flavonoids, which led to the UV-irradiated extracts’ enhanced capabilities to scavenge free radicals [112]. Another study was also able to increase exo- and endo-polyphenols production through application of stimulatory agents, where only 1.0 g/L of the most effective agent, linoleic acid, was found to significantly improve the synthesis of ferulic acid (**108**), gallic acid (**104**), epicatechin-3-gallate (ECG) (**109**), epigallocatechin-3-gallate (EGCG) (**110**), phelligridin G (**111**), inoscavin B (**90**), and davallialactone (**112**) to give rise to more robust scavenging activities of the treated extracts [113]. Tween-20 was also proven to be a potent stimulatory agent for the increase in polyphenols ferulic acid (**108**), naringin (**113**), ECG (**109**), EGCG (**110**) content, which subsequently led to enhanced antioxidant activities of treated products [114].

### Tropicoporus linteus

#### Polysaccharides

A water-soluble heteropolysaccharide was isolated from the mycelia of *Tropicoporus linteus*, with a composition of arabinose, xylose, glucose, and galactose in the molar ratio of 4.0:6.7:1.3:1.0 and a molecular weight of 343,000 kDa, whereby the backbone consisted of α-1,2-, α-1,4, β-1,4-, and β-1,5-glycosydic bonds [115]. The polysaccharide was capable of regulating the mitogen-activated protein kinases (MAPK) and peroxisome proliferator-activated receptor (PPAR) signalling pathways to exert an anti-inflammatory effect. Another high molecular weight polysaccharide (123.8 kDa) that is rich in galacturonic acid (35.7% of monosaccharide composition) with small amounts of arabinose, galactose, xylose, rhamnose, and fucose showed immunostimulatory activities [116].

A lower molecular weight (1,3;1,6)-β-D-polysaccharide (20.7 kDa) consisting mainly of glucose (78.88%) mannose (8.32%) and galactose (8.06%) was also obtained from *T. linteus* [117]. It was reported to inhibit TNF-α and IL-6 release in RAW264.7 cells, while increasing IL-10 levels, thus suggesting a possible role in the restoration of the IL6/IL10 balance. Another polysaccharide with a molecular weight of 15.5 kDa is also mainly made up of glucose (53.2%), mannose (14.9%), and galactose (13.5%) with 5.5% composition of (1,3;1,6)-β-d-glucans, where it was found to be capable of inhibiting NO production in RAW264.7 cells [118].

Three antioxidant polysaccharides, PL-W, PL-A, and PL-N, were extracted from the mycelia of *T. linteus*, where PL-W was composed of glucose and mannose (molar ratio 8:1), PL-A composed of glucose, mannose, xylose, and arabinose (molar ratio 8:1:1:1), while PL-N consists of xylose, arabinose, glucose, and galactose (7.8:5.5:1.8:1.0) [119]. All three polysaccharides were soluble in water at 10 g/L and possess potent antioxidant capacities in a concentration-dependent manner. This is in agreement with the findings of Yan et al. [120], where PL-N was found to have antioxidant activities, which can be enhanced by ultrasonic treatment. Besides that, two novel heteropolysaccharides, PLP1-I and PL-A11, with molecular weights of ∼290,000 kDa and 13.8 kDa respectively, were obtained from *T. linteus* mycelia [121, 122]. PLP1-I consists of only glucose and galactose (molar ratio 8.9:1.0) with a 1,4-α-d-glucopyranose backbone, while PL-A11 is composed of arabinose, xylose, mannose, and glucose (molar ratio 1.1:1.3:1.0:6.6) with a 1,4-α-d-glucopyranosyl, 1,2-α-d-xylopyranosyl, and 1,3-α-d-arabinofuranosyl backbone. Nevertheless, both polysaccharides were found to increase antioxidant enzyme activities in a dose-dependent manner. Another two polysaccharides with antioxidative activities, PLPS and C-PLPS, was recently isolated, where both contained arabinose, xylose, mannose, glucose, and galactose in molar ratios of 1.0:1.8:3.8:40.1:1.4 and 1.0:1.5:3.4:25.2:1.1 respectively [123].

A branched-type glycan with an average molecular weight of 3172.9 kDa, pyranoid sugar ring conformation, and α- and β-linkages was found to be capable of exerting an anti-diabetic effect by reducing blood glucose levels in diabetic mice [124]. This agrees with the findings of Liu et al. [125] where two *T. linteus* polysaccharides with backbones consisting of repeating α-d-glucose(1,4)-α-d-glucose(1,6) units (PLPS-1) and 1,3-α-d-glucose and 1,6-α-d-glucose (PLPS-2) respectively were observed to ameliorate insulin resistance.

A thorough evaluation of PLPS-1 and PLPS-2 previously conducted by Mei et al. [126] revealed that PLPS-1 consisted of glucose, arabinose, fucose, xylose in a molar ratio of 21.964:1.336:1.182:1:1, while PLPS-2 consisted of glucose, galactose, mannose, arabinose, fucose, xylose in a molar ratio of 14.368:2.594:1.956:1.552:1.466:1, where PLPS-1 was found to possess antitumor activities against S180 sarcoma cells. Another 343,000 kDa polysaccharide was reported to exert antitumor activities against HepG2 cells [127] and a hepatoprotective effect in mice [128], where structural characterization revealed the presence of seven glycosidic residues Araf (1 → , → 5) Araf (1 → , → 4) Xylp (1 → , → 2) Xylp (1 → , → 2,4) Xylp (1 → , → 4) Glcp (1 → , and → 4,6) Galp (1 → in the molar ratio of 2.28:1.83:3.17:2.64:1.03:1.36:1.00.

A polysaccharide composed of glucose, galactose, mannose, fucose, and xylose in a molar ratio of 4.36: 2.34: 2.09: 0.88: 0.42 showed prebiotic potentials by increasing amounts of beneficial bacteria, such as *Bacteroides*, *Prevotella*, and *Butyricimonas*, and reducing pathogenic bacteria including *Escherichia*, *Shigella*, *Morganella*, and *Intestinimonas* [129]. Besides that, a polysaccharide with a monosaccharide composition of fucose, rhamnose, arabinose, glucuronic acid, galactose, glucose, and xylose (1.4:0.5:0.9:1.6:4.7:84.8:6.0) exerted bacterio-static activities against bacteria *Staphylococcus aureus*, *Escherichia coli*, and *Bacillus subtilis*, demonstrating the potential of *T. linteus* polysaccharides for clinical applications [130].

#### Terpenes and terpenoids

Phellilane L (**114**) is a cyclopropane-containing sesquiterpenoid isolated from *T. linteus* that was found to possess antimicrobial activities against *P. gingivalis* [131]. A γ-Ionylidene-type sesquiterpenoid obtained from *T. linteus*, (-)-*trans*-γ-monocyclofarnesol (**115**), also exhibited antimicrobial effects on *P. gingivalis* [132], while phellidene E (**116**), ( +)-γ-ionylideneacetic acid (**117**) [132] and phellilane H (**118**) [133] did not show any detectable activities. Besides that, phellinulins A-N (**119–132**) were extracted from the mycelium of *T. linteus*, where phellinulins A (**119**), H (**126**), I (**127**), K (**129**), and M (**131**) were found to have hepatoprotective properties [134].

#### Phenolic compounds

Phellifuropyranone A (**133**) and phelligridin G (**111**) can be found in *T. linteus*, and possess in vitro antiproliferative activity against mouse melanoma and human lung cancer cells [135]. Several other polyphenols, including hispidin (**134**), phelligridimer A (**135**), davallialactone (**112**), methyldavallialactone (**136**), hypholomine B (**137**), interfungin A (**138**), inoscavin A (**139**), protocatechuic acid (**97**), protocatechualdehyde (**95**), caffeic acid (**99**), and ellagic acid (**140**) were also isolated by Lee et al. [136], where davallialactone (**112**), hypholomine B (**137**), and ellagic acid (**140**) were capable of inhibiting aldose reductase to reduce diabetic complications. In addition, davallialactone (**112**), hispidin (**134**), hypholomine B (**137**), and caffeic acid (**99**) were found to have antioxidant activities [137], while hispidin (**134**) and hypholomine B (**137**) also exerts hypolipidemic and hepatoprotective effects [138].

In agreement with Min et al. [137] and Chiu et al. [138], hispidin (**134**) have been demonstrated to be a potent antioxidant by various studies, thus providing protection against oxidative stress [139–142]. Due to the significant bioactivities and potential applications of hispidin, methods have been developed to enhance the production of hispidin, such as through solid-state fermentation using pearl barley medium [143].

Another notable polyphenol that has been detected in *T. linteus* is hispolon (**141**) [144]. Hispolon has been reported to have various pharmacological activities, such as anti-inflammatory properties, [145–148], antioxidant activities [149], and antitumor effects [150–153].

A catechol-containing phenylpropanoid derivative found in *T. linteus*, 3,4-dihydroxybenzalacetone (Osmundacetone) (**93**), has been shown to attenuate inflammation [154], exert antioxidant activities [155], suppress the metastasis and formation of cancer [156], and protect against aging-associated myocardial alterations [157]. Osmundacetone (**93**) and inotilone (**142**), have also been isolated from *T. linteus*, where they have been demonstrated to have antioxidant properties [158] and inhibit neuraminidase activity to thus exert an antiviral effect [13].

## Health-promoting properties

### Taiwanofungus camphoratus

#### Anticancer

**Fig. 3:**
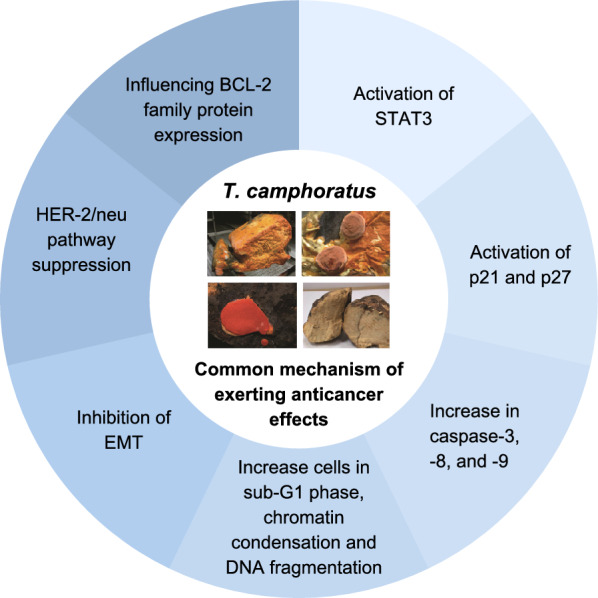
Mechanisms found to be employed by *Taiwanofungus camphoratus* to exert anticancer effects

*T. camphoratus* possesses a wide range of health-benefiting properties, which has been summarized in Table 2, whereby its anticancer properties are probably the most extensively studied aspect (Fig. 3). The ethanolic extract of *T. camphoratus* have been shown to exert anticancer effects on SMMC-7721 and HepG2 cells through inhibiting the activation of signal transducer and activator of transcription 3 (STAT3) [159], and B16-F0 cells via inducing cell cycle arrest and apoptosis [160]. Additionally, *T. camphoratus* ethanolic extracts also induced cell cycle arrest and suppressed the growth of Hep 3B and Hep J5 cells, where caspase-3 and cell cycle inhibitors p21 and p27 were also activated in the cells [161]. Three ergostane-type triterpenes isolated from the fruiting bodies of *T. camphoratus* caused a significant increase of HT-29 (Colon cancer) cells in the sub-G1 phase from the control value of 0.77% to about 41%, 32% and 29% respectively (Compound 1, 2 and 3), strongly indicating the ability of these triterpenes to induce apoptosis in HT-29 cells [162].

**Table 2:** Health-promoting properties of *Taiwanofungus camphoratus* extracts and bioactive compounds

Health-promoting property	Sample	Study model/assay	Finding/observation	References
Anticancer	Ethanol extract	SMMC-7221 and HepG2 cells	Inhibition of STAT3	Zhu et al. [159]
	Ethanol extract	B16-F10 cells	Cell cycle arrest and apoptosis	Wang et al. [160]
	Ethanol extract	Hep3B and HepJ5 cells	Cell cycle arrest and growth suppression	Lin et al. [161]
	Ergostane-type triterpenes	HT-29 cells	Increase of cells in the sub-G1 phase from the control value of 0.77 to about 41% (Compound 1), 32% (Compound 2) and 29% (Compound 3)	Yeh et al. [162]
	Fermented culture broth	MDA-MB-231 cells	Epithelial-mesenchymal transition inhibition	Hseu et al. [163]
	Fermented culture broth	SKOV-3 cells	Suppression of the HER-2/neu signaling pathway	Yang et al. [164]
	Fermented culture broth	MCF-7 cells	Dose- and time-dependent series of apoptotic events, as evidenced by accumulation of cells in the sub-G1 phase, chromatin condensation, internucleosomal DNA fragmentation and loss of cell viability	Yang et al. [165]
	Crude extract	HeLa and C-33A cells	Increased the activities of caspase-3, -8, and -9 in nd led to a dose-dependent rise in cytosolic cytochrome	Yang et al. [166]
	Crude extract	SKOV-3 and TOV-21G cells	Caspase 3, 8, 9 and cytochrome c increased	Liu et al. [167]
	Crude extract	T24 cells	Susceptible at 50 µg/mL with about 50% of the cells inhibited at the 72 h time-point	Peng et al. [168]
	Ethyl acetate extract	Hep 3B cells	Calpain and caspase-12 activation due to increase of cytoplasmic calcium (Ca2 +)	Kuo et al. [169]
	Ethyl acetate extract	Hep G2 and PLC/PRF/5 cells	Marked increase in apoptotic promoting factors	Hsu et al. [170]
	Methanol extract	Hep 3B and HepG2 cells	72 h treatment recorded that about 40% of the Hep 3B cells and about 98% of the Hep g2 have undergone apoptosis	Song. et al. [171]
	Methanol extract	Jurkat cells	IC50 value of approximately 40 µg/mL	Rao et al. [172]
	Solid-state cultured broth	C3A and PLC/PRF/5 cells	Synergistic antiproliferative effects when administered in combination with cisplatin and mitomycin	Chang et al. [173]
Hypoglycemic	Antcin K	C2C12 cells	Enhance membrane glucose transporter 4 (GLUT4) and phosphor-Akt expressions	Kuo et al. [174]
	Eburicoic acid	High-fat diet-induced C57BL/6J mice	Increase AMPK phosphorylation, Akt, and membrane GLUT4 level	Lin et al. [175]
	Sulphurenic acid	Streptozotocin-induced diabetic C57BL/6J mice	Rise of insulin levels and reduction in the concentration of glycated hemoglobin (HbA1c)	Lin et al. [176]
	Dehydroeburicoic acid	C2C12 cells; Streptozotocin-induced diabetic C57BL/6J mice	Higher expression of GLUT4 in vitro 43.6–46.5% decrease in blood glucose level in vivo	Kuo et al. [177]
Hypolipidemia	Antcin K	Streptozotocin-induced diabetic C57BL/6J mice	Fatty acid synthase (FAS) expression levels declined while hepatic expression levels of carnitine palmitoyl transferase 1a (CPT-1a) and peroxisome proliferator-activated receptor α (PPARα) rose	Kuo et al. [178]
	Eburiocoic acid and Sulphurenic acid	High-fat diet-induced C57BL/6J mice	Increased expression of PPAR-α in the liver which facilitated fatty acid oxidation and the reduction of lipid levels	Lin et al. [175]
	Ergostatrien-3β-ol	High-fat diet-induced C57BL/6J mice	Reduced visceral adipocyte size and induced hepatic ballooning degeneration	Kuo et al. [178]
Antioxidant	Ethanol extract	DPPH and ROS scavenging assay	The activity at 4 mg/mL was around 83%, almost as high as that of vitamin C at 0.5 mg/mL, which demonstrated around 88% activity in DPPH assay The activity at 4 mg/mL was around 71–87%, higher than that of vitamin C at 0.5 mg/mL, which demonstrated around 67% activity in SOD scavenging assay	Wang et al. [160]
	Crude oil and polysaccharide	DPPH assay	EC50 values of 47 µg/mL and 22 µg/mL respectively, significantly than that of tert-Butylhydroquinone (TBHQ) and ascorbic acid at 11.2 µg/mL and 6 µg/mL respectively	Zhang et al. [179]
	Mycelium	DPPH assay and phenolic compound evaluation	Strong and dose-dependent scavenging activity with a phenolic content of 20 mg/g	Hsieh et al. [14]
	Selenium rich polysaccharides	DPPH and ABTS assay	Selenium enrichment almost doubled the DPPH and ABTS radical scavenging activity	Li et al. [35]
Hepatoprotective	Antroquinonol	Oxidative stress-induced HepG2 cells	Antroquinonol pretreatment considerably and dose-dependently decreased the rise in ALT and AST caused by ethanol after 24 h in HepG2 cells	Kumar et al. [180]
	Mycelium	Oxidative stress-induced ICR mice model	Ethanol-induced hepatic enzyme levels were notably and dose-dependently decreased by pretreatment, with AST being more sensitive to the treatment	Kumar et al. [180]
	Antroquinolol	Obesity-induced Wistar rats	Exerts hepatoprotective effects by inhibiting hepatic lipid accumulation	Chen et al. [181]
	Antrosterol	Chronic alcohol-induced liver disease in C57BL/6J (B6) mice	Reduction in serum/hepatic lipids, rise in output of fecal lipid/bile-acid output, amplified hepatic antioxidant activities, reduced serum alcohol level, decrease liver inflammation, fibrosis, serum AST/ALT and TNF-α/IL-1β	Chang et al. [182]
Anti-inflammatory	Ethanol extract	LPS-stimulated RAW264.7 cells and Dextran sulfate sodium-induced C57BL/6J mice	Inhibition of nitric oxide (NO), prostaglandin (PG) E2, inducible nitric oxide synthase (iNOS) and cyclooxygenase-(COX-)2, induction of p38-MAPK, extracellular signal-related kinases (ERK), and nuclear factor kappa B (NF-κB) in vitro Similar findings in vivo with reduction in TNF-α and IL-6 mRNA expression levels in treated groups	Park and Park [183]
	Ergostatrien-3β-ol	Ultraviolet B-irradiated BALB/cAnN.Cg-Foxn1nu/CrlNarl hairless mice	Significant inhibition of metalloproteinase-1 (MMP-1), IL-6, iNOS and NF-κB in skin	Kuo et al. [184]
	Methanol extract of wood-cultured fruiting bodies and solid-state-cultured mycelia	Skin-flap ischemia damage-induced FVB/NJNarl mice	Significant lowered the skin flap's necrosis and inflammatory cell infiltration in both the sub-dermis and epidermis Decrease in iNOS, IL-6, TNF-α, and NF-κB genes	Tsai et al. [185]
	Acetylantroquinonol B	Polyinosinic-polycytidylic acid (poly IC)-induced RAW 264.7 cells	Potent inhibitor of NO production with an IC50 of 0.57 ± 0.06 µM	Tu et al. [186]
	Benzoids	LPS-stimulated RAW264.7 cells	Dose-dependent suppression of NO production	Chen et al. [187]
Antiviral	Ethanol extract	Herpes simplex virus type 1 and 2 (HSV-2)-infected Vero cells	Significantly inhibited viral replication between 1–4 h upon infection of Vero cells	He et al. [11]
	Ethanol extract	Dengue virus-infected Meg-01 cells	Pre-treatment inhibited replication in Meg-01 cells by increasing the expression of antiviral cytokine interferon (IFN)-α and decreasing the release of pro-inflammatory cytokines interleukin (IL)-6 and IL-8	Chen et al. [188]
	Polysaccharides	Hepatitis B virus (HBV)-producing cell line MS-G2	Significantly inhibited hepatitis B e antigen (HBeAg) synthesis (30% inhibition at 50 µg/mL)	Lee et al. [189]
	Atrodins	Hepatitis C virus (HCV) protease assay	Antrodins A to E, except antrodin B, exhibited potent inhibitory effects against the HCV protease, with Antrodin A having the highest potency with a half-maximal inhibitory concentration (IC50) value of 0.9 µg/mL	My et al. [190]

Furthermore, triple-negative breast cancer (MDA-MB-231) cells were also responsive to the anticancer effects of the fermented culture of *T. camphoratus*, through an inhibition of the EMT, further suggesting the potential of *T. camphoratus* as an anticancer agent [163]. Moreover, Yang et al. [164] reported that the fermented culture of *T. camphoratus* also produced inhibitory effects against SKOV-3 human ovarian carcinoma cells via suppression of the HER-2/neu signalling pathway. Besides, the fermented culture of this mushroom extract also exerted a dose- and time-dependent series of apoptotic events in MCF-7 cells, as evidenced by the accumulation of cells in the sub-G1 phase, chromatin condensation, internucleosomal DNA fragmentation, and loss of cell viability [165].

The crude extract of the fruiting body of cultivated *T. camphoratus* induced cytotoxicity on HeLa and C-33A human cervical cancer cell lines, with higher potency recorded on C-33A in comparison to HeLa cells [166]. According to the results of the caspase activity assay, the crude extract significantly increased the activities of caspase-3, -8, and -9 in HeLa and C-33A cells and led to a dose-dependent rise in cytosolic cytochrome in the cytochrome c assay, thus indicating that apoptosis involves both intrinsic and extrinsic signalling pathways [166]. Similar effects were also exerted by the crude extract on SKOV-3 and TOV-21G ovarian cancer cells where the activity of caspases-3, -8, -9 and cytochrome c were found to be increased [167]. Bad protein from the Bcl-2 family was observed to be increased in SKOV-3 cells while the expression of Bim and Bak proteins increased and Bcl-xL decreased in TOV-21G cells. Moreover, T24 bladder cancer cells were also found to be susceptible to the crude extract of *T. camphoratus* with a concentration of about 50 µg/mL with about half of the cells in the control being inhibited at the 72 h time-point [168].

The ethyl acetate extract of *T. camphoratus* was reported to be effective in the liver cancer cell line Hep 3B via the increase of cytoplasmic calcium (Ca^2+^) and subsequent induction of calpain and caspase-12 activation [169], as well as HepG2 and PLC/PRF/5 cells, as evidenced by the marked increase in apoptotic promoting factors [170]. In addition, Hep3B and HepG2 cells were also susceptible to the methanolic extract of *T. camphoratus*, where treatment for 72 h resulted in apoptosis of around 40% of the Hep3B cells and 98% of the HepG2 [171]. Furthermore, Rao et al. [172] reported that methanol extract also inhibited Jurkat leukaemia cells with IC_50_ value of approximately 40 µg/mL.

The solid-state cultured broth of *T. camphoratus* also managed to exert synergistic antiproliferative effects in C3A and PLC/PRF/5 hepatoma cells when administered in combination with cisplatin and mitomycin via the inhibition of the multidrug resistance (MDR) gene expression and the cyclooxygenase-2 (COX-2)-dependent inhibition of phospho-protein kinase B (p-Akt), which in turn resulted hepatoma cells to undergo apoptosis [173].

#### Hypoglycemic and hypolipidemic

*T. camphoratus* is also known for its antiglycemic effects, where antcin K from the fruiting body of *T. camphoratus* can enhance membrane glucose transporter 4 (GLUT4) and p-Akt expressions in vitro in C2C12 myotube cells and induce an antihyperglycemic effect (40 mg/kg/day) comparable to Metformin (300 mg/kg/day) in vivo in C57BL/6J mice [174]. Another compound, eburicoic acid TRR, from *T. camphoratus* was able to increase AMPK phosphorylation, Akt, and membrane GLUT4 level in skeletal muscles, suggesting its capability to produce antihyperglycemic effects via an insulin-dependent pathway or possibly through activation of AMPK in skeletal muscles [175]. Furthermore, the administration of sulphurenic acid obtained from *T. camphoratus* resulted in the rise of insulin levels in streptozotocin-induced C57BL/6J diabetic mice, possibly due to an increase in pancreatic Langerhans islets’ size and number upon treatment and caused a notable reduction in the concentration of glycated hemoglobin (HbA1c) [176]. It was noted that sulphurenic acid stimulates the phosphoinositide 3-kinase (PI3K)/Akt pathway, which suppresses hepatic glucose production to result in a hypoglycemic effect. Kuo et al. [177] also reported that dehydroeburicoic acid from *T. camphoratus* increased the expression of GLUT4 in C2C12 myotube cells and also resulted in 43.6–46.5% decrease in blood glucose level in Streptozotocin-induced C57BL/6J diabetic mice.

Antcin K from *T. camphoratus* has also been found to exert a hypolipidemic effect by decreasing the expression of fatty acid synthase levels and increasing the hepatic expression levels of carnitine palmitoyltransferase 1A (CPT-1A) and PPAR-α [174, 176]. Eburicoic acid and sulphurenic acid derived from *T. camphoratus* also increased the expression of PPAR-α in the liver of high-fat diet (HFD)-induced C57BL/6J mice, thus facilitating fatty acid oxidation and reduction of lipid levels [175, 176]. In addition, Kuo et al. [178] reported that Ergostatrien-3β-ol from *T. camphoratus* reduced visceral adipocyte size and induced hepatic ballooning degeneration. All four above-mentioned compounds were also found to SREBP-1C to downregulate transcription of genes responsible for fatty acid synthesis, suggesting the potent hypolipidemic effects of *T. camphoratus *[174, 175, 178, 191].

#### Antioxidant

Ethanolic extract of *T. camphoratus* fruiting body displayed strong DPPH radical and superoxide dismutase (SOD)-like scavenging activities at levels nearly equivalent and stronger than vitamin C respectively [9]. In DPPH assay, the activity at 4 mg/mL of the extract was around 83%, almost as high as that of vitamin C at 0.5 mg/mL, which demonstrated around 88% activity. In contrast, the extract’s activity at 4 mg/mL in the SOD scavenging assay was around 71–87%, higher than that of vitamin C at 0.5 mg/mL, which demonstrated around 67% activity. The potent antioxidant activities of *T. camphoratus* were further highlighted in another study where *T. camphoratus* crude oil and polysaccharide had half maximal effective concentration (EC_50_) values of 47 µg/mL and 22 µg/mL respectively in DPPH assay, significantly than that of tert-Butylhydroquinone and ascorbic acid at 11.2 µg/mL and 6 µg/mL [179]. Besides that, *T. camphoratus* mycelium extract recorded strong and dose-dependent scavenging activity, which can be attributed to its high phenolic content, observed to be at 20 mg/g [14]. Furthermore, Li et al. [35] reported that the polysaccharides isolated from *T. camphoratus* which were enriched with selenium recorded almost double the DPPH and ABTS radical scavenging activity in comparison to the unenriched polysaccharides, thus suggesting the possibility of raising the antioxidant properties of *T. camphoratus* through modifications of isolated bioactive compounds.

#### Hepatoprotective

Kumar et al. [180] reported that treatment with antroquinonol and mycelia of Golden-*T. camphoratus* displayed effective protection of the liver against alcohol-induced damage both in vitro and in vivo, whereby both antroquinonol and mycelia pretreatment considerably and dose-dependently decreased the ethanol-associated rise in alanine aminotransferase (ALT) and aspartate transaminase (AST) after 24 h in oxidative stress-induced HepG2 cells [180]. Furthermore, Chen et al. [181] revealed the potential of antroquinonol from Golden-*T. camphoratus* to inhibit hepatic lipid accumulation and release of inflammatory cytokines through the upregulation of hepatic adenosine diphosphate (ADP)-activated protein kinase and concurrent downregulation of SREBP1 expression and fatty acid synthase expression.

Hepatoprotective activities have also been found in antrosterol from *T. camphoratus*, whereby pre-treatment of chronic alcohol liver disease-induced mice could reduce serum/hepatic lipids, increase fecal lipid/bile-acid output, amplify hepatic antioxidant activities, reduce serum alcohol level, and decrease liver inflammation, fibrosis, serum AST/ALT, and TNF-α/IL-1β [182]. These combined findings provide valuable insights into the multifaceted mechanisms underpinning the hepatoprotective role of compounds isolated from *T. camphoratus*.

#### Anti-inflammatory

The ethanol extract of *T. camphoratus* grown on germinated brown rice was reported to inhibit NO, prostaglandin E2 (PGE2), iNOS and COX2 production while inducing p38-MAPK, extracellular signal-related kinases (ERK) and NF-κB in LPS-stimulated RAW264.7 cells [183]. The same study also reported similar observations for the in vivo analysis in addition to the reduction of TNF-α and IL-6 mRNA expression levels in treated dextran sulfate sodium (DSS)-induced mice. Similar findings were also reported by Kuo et al. [184], in which significant inhibition of MMP-1, IL-6, iNOS, and NF-κB were observed in the skin of ultraviolet B-induced mice for topical administration of 25 and 100 μM ergostatrien-3β-ol isolated from *T. camphoratus*.

Besides, Tsai et al. [185] reported a decrease in gene expressions of iNOS, IL-6, TNF-α and NF-κB along with significantly lowered necrosis and inflammatory cell infiltration (both sub-dermis and epidermis) in skin-flap ischemia damage-induced mice treated with the methanol extract of the wood-cultured fruiting body and solid state-cultured mycelia of *T. camphoratus*. In addition, acetylantroquinonol B [186] and benzoids [187] were also found to inhibit the NO production in inflammation-induced RAW 264.7 cells, further demonstrating the anti-inflammatory capabilities of *T. camphoratus*.

#### Antiviral

A limited amount of research on the antiviral effects of *T. camphoratus* has been conducted over the past decade. According to He et al. [11], a fraction of the crude ethanol extract of *T. camphoratus* exhibited notable antiviral activity against the herpes simplex virus type 1 (HSV-1) and herpes simplex virus type 2 (HSV-2), significantly inhibiting the viral replication between 1–4 h upon infection of Vero cells. However, antiviral activity reduced with the addition of the extract after 5 h of infection, suggesting low inhibition of late multiplication of virus [11]. In addition, Chen et al. [188] reported that the pre-treatment of two ethanolic extracts of *T. camphoratus* inhibited the replication of Dengue virus in Meg-01 cells by increasing the expression of antiviral cytokine interferon alpha (IFN-α) and decreasing the release of pro-inflammatory cytokines IL-6 and IL-8.

Polysaccharides from *T. camphoratus* were also found to possess anti-Hepatitis B virus (HBV) activity, where polysaccharides from B86 and 35,398 strains most significantly inhibited hepatitis B e-antigen (HBeAg) synthesis, with about 30% antiHBeAg inhibition percentage for both strains at a concentration of 50 µg/mL [189]. Apart from HBV, antrodins A to E, except antrodin B, isolated from *T. camphoratus* exhibited potent inhibitory effects against the HCV protease, whereby antrodin A had the highest potency with a half-maximal inhibitory concentration (IC_50_) value of 0.9 µg/mL [190].

### Inonotus obliquus

#### Anticancer

*I. obliquus* is capable of exerting a wide range of therapeutic properties, as summarized in Table 3, which includes its anticancer activities. *I. obliquus* has been used in folk medicine for the treatment of cancer [4], including its use to heal lip tumours [192]. In the twentieth century, the remarkable ability of *I. obliquus* to reduce the number of cancer cases in the population was noticed by the Soviet health authorities [40], leading to increasing amounts of scientific research to be conducted on this medicinal mushroom (Table 3).

**Table 3:** Health-promoting properties of *Inonotus obliquus* extracts and bioactive compounds

Health-Promoting Property	Sample	Study model/assay	Finding/Observation	References
Anticancer	Aqueous extract	B16-F10 cells	51% reduction in cell proliferation was recorded after a 48-h exposure to 750 μg/mL of extract	Youn et al. [195]
	Aqueous extract	HT-29 cells	Dose-dependent inhibition with apoptosis, maximum inhibition of 56% at 1.0 mg/mL for 48 h treatment	Lee et al. [196]
	Aqueous extract	3LL carcinoma-implanted C57BL/6 mice	Consistent intake decreased 25% of the metastatic tumor nodules and reduced 60.3% (Day 16) of tumor growth in comparison with the control group	Arata et al. [6]
	Aqueous extract	DLD-1 inoculated BALB/c mice	33% inhibition in tumour growth at the end of 36 days for a concentration of 7 mg/mL	Yuan et al. [197]
	Ethanol extract	HT-29 cells	Cell cycle arrest at G1 phase, induced higher levels of p21 and p27, resulting in the inhibition of cyclin-dependent kinase (CDK) and reduced Rb phosphorylation	Lee et al. [71]
	Ethanol extract	NCI-H460 cells	44.2 to 74.6% inhibitory rate with extract from cultivated sclerotia having the highest inhibition rate	Sun et al. [199]
	Ethanol and hot water extracts	DLD-1 cells	Induction of apoptosis by reducing oxidative stress	Hu et al. [200]
	Methanol extract	IMR-90, A549, PA-1, U937 and HL-60 cells	Fruiting body extract—PA-1 = HL-60 > U937 > A549 >vIMR90 Sclerotium extract—PA-1 > U937 = HL-60 ≥ A549 = IMR90 Sclerotium more cytotoxic to all cancer cells lines	Nakajima et al. [201]
	Polysaccharide	LLC1 cells	Inhibitory effects in both time and dose-dependent manners	Jiang et al. [92]
	ISP2a polysaccharide	SGC-7901 tumour-bearing nude mice	100 mg/kg of IPS2a in SGC-7901 tumour-bearing mice caused more than 50% inhibition in tumour growth	Fan et al. [202]
	3β-hydroxylanosta-8,24-dien-21-al	A549, H1264, H1299 and Calu-6 cells	IC50 values of 85.3 μg/mL (A549), 90.9 μg/mL (H1264), 128.0 μg/mL (H1299) and 75.1 μg/mL (Calu-6)	Baek et al. [203]
	Aqueous extract	HCT-15 cells	Dose-dependent inhibition in growth via induction of apoptosis	Cha et al. [194]
Hypoglycemic	Polysaccharides	Streptozotocin-induced diabetic Kunming mice	Concentration of 900 mg/kg significantly improved glucose tolerance, reduced fasting blood glucose levels, increased hepatic glycogen levels, and alleviated insulin resistance	Wang et al. [9]
	Methanol extract	C57BKS db/db mice	Improved blood glucose level by modifying intestinal bacteria	Ye et al. [204]
	Water-soluble melanin complex	3T3-L1 adipocytes	Increase in insulin-stimulated glucose uptake, dose-dependent increase in Akt phosphorylation and glucose transporter 4 (GLUT4) translocation	Lee and Hyun [205]
	Fermented powder	Streptozotocin-induced diabetic rates	Significantly (*p* < 0.05) lower glucose level (mg/100 ml serum) in groups with diet supplemented with 50 g/kg fermented chaga power as compared supplementation with non-fermented chaga powder and control	Cha et al. [206]
Hypolipidemia	Polysaccharides	Oleic acid (OA)-Induced HepG2 cells	Decrease total cholesterol (TC), triacylglycerol (TG) and low-density lipoprotein (LDL) levels, increased the high-density lipoproteins (HDL) levels via the activation of AMPK and induction of fatty acid oxidation	Yang et al. [70]
	Methanol extract	C57BKS db/db mice	Decrease LDL-C, TC and TG levels, increase HDL-C level	Ye et al. [204]
	Aqueous extract	High-fat diet-induced C57BL/6 mice	Decrease TC, TG and LDL-C, increase liver glycogen and HDL-C for 250 and 500 mg/kg concentrations	Zhang et al. [208]
	Oligosaccharides	High-fat diet-induced Kunming mice	Hyperlipidemic effects by balancing intestinal flora	Wu et al. [209]
Antioxidant	Isocoumarins	FRAP, DPPH and ABTS assay	Significant antioxidant activity as compared to Trolox	Chang et al. [19]
	Sclerotium and mycelium	Isolated human lymphocytes	Protection of DNA from oxidative damage induced by H_2_O_2_	Park et al. [210]
	Triterpenoids, steroids, and polyphenols	DPPH assay and HaCaT cells	Triterpenoids and steroids had fairly strong antioxidant activity while polyphenols possessed significant antioxidative capabilities and protected HaCaT cells from H_2_O_2_-induced oxidative stress	Cui et al. [211]
	Polysaccharides	Hydroxyl radical scavenging, Superoxide anion scavenging and FRAP assay	Stronger antioxidant activity in polysaccharides with higher uronic acid and proteinous substance content	Huang et al. [212]
	Polysaccharide	DPPH and hydroxyl radical scavenging assay	Scavenging activity in DPPH of 82.3% and hydroxyl radicals of 81.3% at 5 mg/mL	Hu et al. [213]
	Polysaccharides	Diethyldithiocarbamate-induced chronic pancreatitis mice	Increased SOD and reduced MDA levels	Hu et al. [213]
Hepatoprotective	Polysaccharides	*Toxoplasma gondii*-induced liver injury in BALB/c mice	Decreased ALT, AST, MDA and NO levels, increased SOD and GSH	Xu et al. [214]
	Aqueous extract	Tert-butyl hydroperoxide-induced oxidative liver injury in primary cultured hepatocyte	Significant protection to hepatocytes even at low concentration of 10 μg/mL	Hong et al. [215]
	Melanin	Chang liver cells; Carbon tetrachloride (CCl_4_) liver damage induced-Sprague Dawley rats	Concentrations of 10^−5^ and 10^−3^ g/L caused 2–2.5 times increase in viability of the liver cells intoxicated with *d*-galactosamine Decrease in fat buildup, steatosis, necrosis, and normalization of serum cholinesterase, gammaglutamyl transpeptidase, total protein and unconjugated bilirubin levels	Parfenov et al. [216]
Anti-Inflammatory	Ethanol extract	LPS-stimulated RAW 264.7 cells	Inhibit proinflammatory mediators which include NO, PGE₂, iNOS, COX-2, TNF-α, IL-1β, and IL-6	Debnath et al. [217]
	Petroleum ether and ethyl acetate extract	LPS-stimulated RAW264.7 cells	Approximately 84.6% (Petroleum Ether) and 78.2% (Ethyl Acetate) reduction in NO production Approximately 96.9% (Petroleum Ether) and 96.6% (Ethyl Acetate) reduction in NF-κB luciferase activity	Ma et al. [101]
	Methanol extract	Histamine-inflammation induced-C57BL6 mice	> 90% suppression of histamine-induced TNF-α	Javed et al. [218]
Antiviral	Aqueous extract	Hepatitis C virus and SPEV cells	High antiviral effect with low cytotoxicity at 0.1 TCD50/cell	Shibnev et al. [219]
	Aqueous and water-ethanol extracts	HIV-1 and MT-4 cells	Antiviral effects at 5.0 μg/mL	Shibnev et al. [12]
	Polysaccharides	FIV, FCV, FPV, FHC-1 and FIPV-propagated CRFK or MDCK cells	Interferes with the virions and/or cell receptors, thus preventing the viral entrance into the cell	Tian et al. [224]
	Aqueous extract	SARS-CoV-2 infected-Vero E6 and Vero cells	Significant binding affinity with the viral S1-carboxy-terminal receptor-binding domain	Teplyakova et al. [220]
	Alcohol-water pilat extract	Herpes simplex virus type-1 (HSV1)-infected Vero cells	Significant anti-HSV-1 activity at concentration from 4.5 × 10^−5^ to 7.5 × 10^0^ mg/mL	Kapp et al. [222]
	Aqueous extract	Herpes simplex virus type-2 (HSV-2)-infected albino mice	90% of mice survived upon prior intraperitoneal administration of 0.4 to 2 mg per mouse	Razumov et al. [223]

Aqueous extracts obtained from the fermentation of *I. obliquus* displayed a dose-dependent inhibition in the growth of HCT-15 cells via the induction of apoptosis [194] and a 51% reduction in B16-F10 melanoma cell proliferation after a 48-h exposure to 750 µg/mL of extract [195]. Further analysis revealed that *I. obliquus* extract may restrict cell proliferation or stimulate differentiation in B16-F10 cells by causing G0/G1 phase arrest and subsequent apoptosis process that occurs when caspase-3 is activated [195]. Another study using aqueous extracts of *I. obliquus* exhibited a dose-dependent inhibition in growth of HT-29 cells via induction of apoptosis, where the highest inhibition of 56% was recorded at the concentration of 1.0 mg/mL at the 48 h time-point. [196]. The anticancer properties of the aqueous extract of *I. obliquus* was also effective in vivo, where a consistent intake of the aqueous extract managed to decrease 25% of the metastatic tumour nodules and reduce 60.3% (Day 16) of tumour growth in 3LL carcinoma-implanted C57BL/6 mice as compared to the control group [6]. Furthermore, Yuan et al. [197] reported that 7 mg/mL of aqueous extract of the fungi exhibited about 33% inhibition in tumour growth in DLD-1 colorectal adenocarcinoma inoculated mice for 36 days.

Apart from aqueous extracts, the ethanol extracts of *I. obliquus* also exhibit promising anticancer effects. According to Lee et al. [198], the extracts stimulated cell cycle arrest at G1 phase, induced higher levels of p21 and p27, resulted in the inhibition of cyclin-dependent kinase (CDK), and consequently reduced retinoblastoma tumour suppressor protein (Rb) phosphorylation. The advancement of the G1 into S cell cycle was halted as a result of these effects, followed by a suppression of HT-29 cell proliferation. Ethanol extracts obtained from wild sclerotium, cultivated sclerotium, and cultivated fruiting body of *I. obliquus* demonstrated anticancer effects with inhibition rates ranging from 44.2 to 74.6% [199]. Moreover, Hu et al. [200] reported that DLD- 1 cells are also susceptible to the ethanol and hot water extracts of *I. obliquus* via the induction of apoptosis and reducing oxidative stress. In addition, in a study conducted by Nakajima et al. [201], methanol extracts from both the fruiting body and sclerotium of *I. obliquus* exhibited anticancer effects on IMR-90, A549, PA-1, U937, and HL-60 cells, whereby PA-1 cells were the most susceptible to both types of methanol extracts, with the methanol extract from sclerotium displaying more potent cytotoxic effects to all tested cancer cell lines.

Other constituents isolated from *I. obliquus* have also demonstrated promising anticancer properties. Polysaccharides isolated from *I. obliquus* were also found to inhibit tumour growth in SGC-7901 tumour-bearing mice [202] and exert anticancer effects in LLC1 cells in both time and dose-dependent manners [92]. Moreover, a triterpenoid named 3β-hydroxylanosta-8,24-dien-21-al induced cytotoxic effects on A549, H1264, H1299, and Calu-6 lung cancer cells with IC_50_ values of 5.3 µg/mL, 90.9 µg/mL, 128.0 µg/mL and 75.1 µg/mL respectively [203]. These findings collectively highlight the potent anticancer effects of a wide variety of compounds derived from *I. obliquus*.

#### Hypoglycemic and hypolipidemic

In addition to its anticancer effects, *I. obliquus* is also known for its hypoglycemic and hypolipidemic activities. Wang et al. [193] showed that oral administration of *I. obliquus* polysaccharides (IOPS) (900 mg/kg) could significantly improve glucose tolerance, reduce fasting blood glucose levels, increase hepatic glycogen levels, and alleviate insulin resistance in streptozotocin induced type 2 diabetes mellitus mice. It was also found that the IOPS increased expressions of PI3K-p85, p-Akt (ser473), and GLUT4 to therefore promote glycolysis and inhibit gluconeogenesis [193]. Furthermore, methanol extracts of *I. obliquus* can significantly improve blood glucose and lipid levels, as well as exert an anti-inflammatory effect in type 2 diabetic mice through downregulation of harmful groups such as *Proteobacteria*, upregulating levels of beneficial bacteria such as *Odoribacter*, and promoting short-chain fatty acid levels by increasing abundance of acid-producing bacteria such as *Alistipes* [204]. Moreover, Lee and Hyun [205] reported that water-soluble melanin complex isolated from *I. obliquus* were found to increase the insulin-stimulated glucose uptake and exert a dose-dependent increase in Akt phosphorylation and GLUT4 translation into the cytoplasm. In addition, another study by Cha et al. [206] mentioned that a diet supplemented with 50 g/kg fermented powder from *I. obliquus* was observed to significantly (p < 0.05) lower the glucose level in 100 mL of serum of streptozotocin-induced type 1 diabetes mellitus mice in comparison with supplementation with non-fermented chaga powder and without any supplementation.

According to Yang et al. [207], the administration of *I. obliquus* polysaccharides was able to decrease the total cholesterol (TC), triacylglycerol (TG), and low-density lipoprotein (LDL) levels, and consequently increased high-density lipoprotein (HDL) levels through the activation of AMPK and induction of fatty acid oxidation. This is consistent with the results obtained from a study conducted by Ye et al. [204] in which mice treated with *I. obliquus* methanol extract exhibited decreased LDL cholesterol (LDL-C), TC and TG levels, and higher HDL cholesterol (HDL-C) levels (p < 0.01). The polysaccharides treatment could also induce a significant reduction in the mRNA expression of SREBP-1, acetyl-CoA carboxylase (ACC), and fatty acid synthase to further contribute to its hypolipidemic activity [207]. Hypolipidemic effects were also observed in the 250 mg/kg and 500 mg/kg *I. obliquus* aqueous extract treatment groups, with decreased TC, TG, and LDL-C and increased liver glycogen and HDL-C in high-fat diet-inducedC57BL/6 mice via the regulation of the PI3K/Akt and AMPK/ACC signalling pathways [208]. Similar hypolipidemic effects were also reported in oligosaccharides of *I. obliquus* via modification of the intestinal bacteria in high-fat fed mice in which the ratio between the population of *Firmicutes* to *Bacteroidetes* faecal flora was reduced in treated groups, reducing the production of fatty acids and thus lowering blood lipids [209].

#### Antioxidant

Two novel isocoumarins found in *I. obliquus* exhibited significant antioxidant activity in FRAP, DPPH and ABTS assays as compared to Trolox, a potent antioxidative [15]. In addition, the sclerotium and mycelium extracts of *I. obliquus* exhibited protection from cellular DNA damage from H_2_O_2_ in healthy human lymphocytes at a similar intensity as natural antioxidants [210]. Another study by Cui et al. [211] found strong antioxidant activities in both the polyphenolic extract and another extract containing triterpenoids and steroids, with more significant activity in the former, suggesting the crucial contribution of phenols to the mushroom’s antioxidative properties.

Five polysaccharides isolated from *I. obliquus* also displayed antioxidant activities, in which stronger antioxidant activities were observed in the ones with higher uronic acid and proteinous substances content [212]. Furthermore, in a study by Hu et al. [213], it was found that 5 mg/mL of IOPS had a scavenging activity of 82.3% in DPPH assay and 81.3% in hydroxyl radical scavenging assay. Additionally, it also elevated SOD levels and reduced malondialdehyde (MDA) levels in a dose-dependent manner, thus bringing levels of oxidative stress close to normal levels in chronic pancreatitis-induced mice, highlighting the potential therapeutic application of *I. obliquus*’ antioxidative functions.

#### Hepatoprotective

**Fig. 4:**
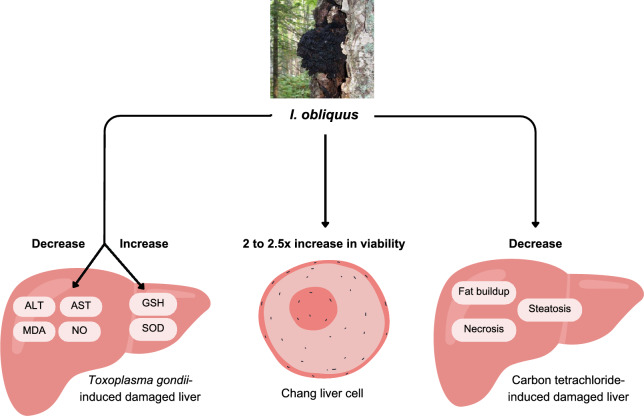
Hepatoprotective properties possessed by *Inonotus obliquus*

A study conducted by Xu et al. [214] investigated the protective effects of polysaccharides isolated from *I. obliquus* in liver damage induced by *Toxoplasma gondii* infection in mice, reported a decrease in ALT, AST, MDA and NO levels, with an increase in SOD and glutathione (GSH). Besides, Hong et al. [215] reported that the aqueous extract of *I. obliquus* resulted in significant protection to tert-butyl hydroperoxide liver damage-induced rat hepatocytes even at a low concentration of 10 µg/mL. Moreover, *d*-galactosamine intoxicated-normal Chang liver cells experienced 2–2.5 times increase in viability upon treatment with 10^−5^ and 10^−3^ g/ L of melanin isolated from aqueous extract of *I. obliquus* [216]. Consistent findings were also obtained in vivo where decrease in fat buildup, steatosis, necrosis, and normalization of serum cholinesterase, gammaglutamyl transpeptidase, total protein and unconjugated bilirubin levels were observed in carbon tetrachloride liver damage induced-Sprague Dawley rats (Fig. 4).

#### Anti-inflammatory

The extract from *I. obliquus* grown on germinated brown rice was found to inhibit proinflammatory mediators NO, PGE2, iNOS, COX-2, TNF-α, IL-1β, and IL-6 in LPS-stimulated RAW 264.7 macrophages [217]. Besides, Ma et al. [101] reported that the petroleum ether extract of *I. obliquus* resulted in approximately 84.6% and 96.9% reductions in NO production and NF-κB luciferase activity respectively in LPS-stimulated RAW 264.7 macrophages, while the ethyl acetate extract stimulated 78.2% and 96.6% reductions in NO production and NF-κB luciferase activity accordingly [101]. Moreover, more than 90% suppression in histamine-induced TNF-α was discovered in histamine inflammation-induced-C57BL/6 mice upon treatment with the methanol extract of *I. obliquus*, thus further highlighting the anti-inflammatory properties of *I. obliquus* [218].

#### Antiviral

Shibnev et al. [219] reported that the infective property of hepatitis C virus was significantly reduced by *I. obliquus* water extracts, which exhibited a high antiviral effect with low cytotoxicity on porcine embryo kidney cells (SPEV), thereby shielding all of SPEV cells post-infection from virus-induced death at a level of 0.1 TCD50/cell. *I. obliquus* water extracts were also able to inhibit the replication of SARS-CoV-2 (nCoV/Victoria /1/2020 strain) in Vero E6 and Vero cell cultures [220], which may be explained by its significant binding affinity with the viral S1-carboxy-terminal of the SARS-CoV-2 receptor-binding domain, producing multivalent hydrogen and non-polar interactions (7.4 to 8.6 kcal/mol) [221].

Aqueous and water-alcohol extracts of *I. obliquus* have also been shown to possess antiviral effects against the human immunodeficiency virus type 1 (HIV-1) at a concentration of 5.0 µg/mL against virus-infected lymphoblastoid cells culture MT-4 [12]. Furthermore, *I. obliquus* alcohol-water pilat extract demonstrated significant in vitro anti-HSV-1 activity at concentrations ranging from 4.5 × 10^–5^ to 7.5 × 10^0^ mg/ml [222]. The observed antiviral property could also be observed in vivo, whereby 90% of HSV-2 infected albino mice survived through prior intraperitoneal administration of 0.4 to 2 mg of *I. obliquus* aqueous extract [223].

In addition, according to Tian et al. [224], IOPS was found to exhibit antiviral effect against a large spectrum of feline viruses, which include the feline influenza virus (FIV), feline calicivirus (FCV), feline panleukopenia virus (FPV), feline herpesvirus 1 (FHV-1) and feline infectious peritonitis virus (FIPV) by interfering with the virions and/or cell receptors, thus preventing the viral entrance into the cell. This suggests the strong antiviral properties of not only crude extracts obtained from *I. obliquus*, but also isolated components such as polysaccharides as demonstrated above.

### Tropicoporus linteus

#### Anticancer

**Fig. 5:**
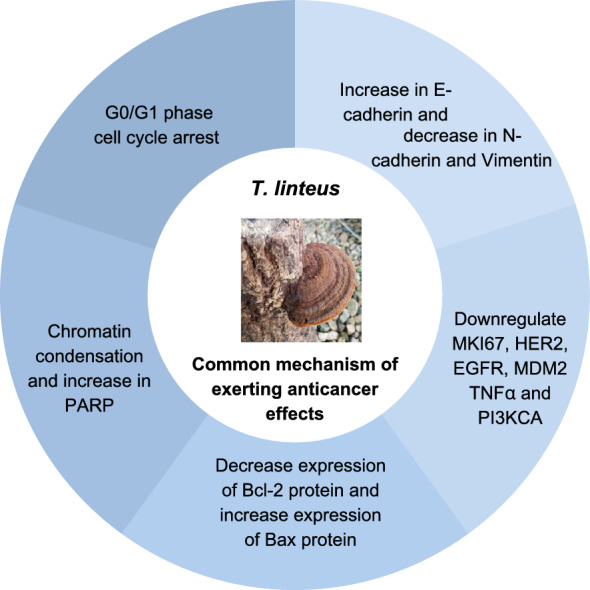
Reported mechanisms through which *Tropicoporus linteus* exert anticancer properties

The anticancer properties of *Tropicoporus linteus* and their underlying mechanisms have been widely studied in recent years (Fig. 5). A comparison between ethanol and water extracts of *T. linteus*, *P. igniarius*, and *P. nigricans* showed that the ethanol extract of *T. linteus* exerts strong cytotoxic effects against cholangiocarcinoma cells, with percent cell inhibition reaching as high as 98.92 ± 0.22 [225]. Besides, the ethanol extract of this mushroom exerted a significant dose-dependent inhibition in MDA-MB-231 cells, where an IC_50_ value of 40 mg/mL was obtained at the 24 h time point [226]. A notable observation is that 30 mg/mL of this extract alone exerted 16.7% inhibition while 10 µg/mL of 5-fluorouracil alone exerted 12.6% inhibition in cell growth, but this value spiked significantly to 53% when both of them were administered together (Table 4).

**Table 4:** Health-promoting properties of *Tropicoporus linteus* extracts and bioactive compounds

Health-promoting property	Sample	Study model / assay	Finding / observation	References
Anticancer	Ethanol extract	KKU-100 & KKU213A cells	Strong cytotoxic effects reaching 98.92 ± 0.22% cell inhibition	Thammavong et al. [225]
	Ethanol extract	MDA-MB-231 cells	Significant dose-dependent inhibition when administered individually 1.8 × synergistic inhibition when used in combination with 5-fluorouracil	Lee et al. [226]
	Ethyl acetate extract	HT-29 cells	Induced apoptosis via induction of cell cycle arrest at G0/G1 phase and DNA fragmentation	Park et al. [227]
	n-hexane layer of ethyl acetate extract	HT-29 cells	Potent anticancer effects with IC50 of 69.8 µg/mL at the 48-h time point	Jeon et al. [228]
	Methanol extract	SW-480 and HCT-116 cells	Increase in anti migratory marker E-cadherin and decrease in pro migratory/pro invasive proteins N-cadherin and Vimentin, with higher selectivity towards SW-480 cells	Šeklić et al. [229]
	Ethanol extract	KRAS-mutated SW480 cells	Increased sensitivity to Cetuximab	Park et al. [230]
	Polysaccharide	S-180 cells	Stronger inhibitory effects than 5-fluorouracil, with up to 93% inhibition at 72 h for 200 µg/mL concentration	Mei et al. [126]
	*P. linteus* fractions PL-ES and PL-I-ES	PC3, DU145, LNCaP, T24, ACHN, A549, MCF-7, AGS, HepG2 and U-87 cells	Anticancer effects as shown in Table 1	Konno et al. [231]
	Hispolon	MCF7, MDA-MB-231, T24 and J82 cells	Suppressed p53 mutant (MDA-MB-231, T24, and J82) and wild-type (MCF7) cells regardless of p53 status Apoptosis characterized by chromatin condensation and poly (ADP-ribose) polymerase (PARP) increase	Lu et al. [232]
	Atractylenolide I	Gastric cancer cachexia patients	Significantly more effective than fish-oil-enriched nutritional supplementation (FOE) at improving appetite, Karnofsky performance status (KPS) and decreasing proteolysis-inducing factor (PIF) positive rates	Liu et al. [233]
Hypoglycemic	Polysaccharide-enriched powder from mycelia	High-fat diet and streptozotocin-induced diabetic Sprague–Dawley rats	Decreased serum glucose, glycated serum proteins, and insulin levels and increased glucokinase and GLUT2 expressions 8 weeks after treatment with 120 and 600 mg/kg	Liu et al. [10]
	Exo-polymers from submerged cultures	Streptozotocin-induced diabetic Sprague–Dawley rats	Aided in pancreatic β-cells repairing	Kim et al. [234]
	Polysaccharide	High-fat diet and streptozotocin-induced diabetic Sprague–Dawley rats	Improved insulin resistance via gut microbiota regulation	Liu et al. [125]
	Polysaccharide	Alloxan-induced diabetic Kunming mice	Mean percentage decrease in blood glucose levels of 22.35%, 16.35%, and 15.19% at concentrations of 100, 200, and 400 mg/kg respectively	Zhao et al. [124]
Hypolipidemia	Polysaccharide-enriched powder from mycelia	High-fat diet and streptozotocin-induced diabetic Sprague–Dawley rats	Significant reduction in triacylglycerides (TG), total cholesterol (TC), free fatty acids (FFA) and low-density lipoprotein (LDL-C) in groups treated with 120 mg/kg and 600 mg/kg	Liu et al. [125]
	Ethyl acetate fraction of mycelia	High fat high fructose diet-induced C57BL/6 mice	Improve body weight, hepatic lipid accumulation and fasting blood glucose via protein regulation	Chiu et al. [138]
	Hispidin and Hypholomine B	Oleic and palmitic acids (O/P) lipid accumulation-induced HepG2 cells	Significant reduction in lipid accumulation	Chiu et al. [138]
Antioxidant	Hispidin	MIN6N beta-cells	10–70 uM had notable reactive oxygen species (ROS) scavenging activity in a dose-dependent manner, with the highest activity being 65%	Lee et al. [235]
	Caffeic acid, inotilone, 4-(3,4-Dihydroxyphenyl)-3-buten-2-one	DPPH, ABTS and FRAP assay	Potent antioxidant activity, with higher antioxidant activity than BHA and Trolox in FRAP assay	Lee et al. [226]
	Polysaccharides	DPPH assay	Activity between 58 to 72% at a concentration of 2 mg/mL	Wang et al. [119]
	Hot water polysaccharide	DPPH assay	Stronger antioxidant activity than ascorbic acid at 5 mg/mL	Kozarski et al. [236]
Hepatoprotective	Polysaccharides	Thioacetamide-induced liver fibrosis in Sprague–Dawley rats	Protective effects via the regulation of the heat shock, oxidative stress, and amino acid and nucleic acid metabolic pathways	Wang et al. [237]
	Hot water extract	Carbon tetrachloride (CCl_4_)-induced liver damage in Sprague–Dawley rats	Reduction of liver peroxidation products to 10% and reduction of cytochrome P450 2E1 (CYP2E1) protein expression to 88%	Jeon et al. [238]
	Methanol extract	H_2_O_2_-injured primary rat hepatocytes from male Wistar rats	Decrease in glutamic pyruvic transaminase released	Kim, Lee, et al. [239]
	Ethyl acetate fraction from methanol extract	Galactosamine-injured primary rat hepatocytes from male Wistar rats	Protections of 68.9 ± 5.3% in H2O2-injured hepatocytes and 46.8 ± 3.9% in galactosamine-injured hepatocytes Maintained glutathione level and RNA synthesis	Kim, Lee, et al. [239]
Anti-Inflammatory	Polysaccharides	Dextran sulfate sodium (DSS) colitis-induced ICR mice	Substantial reduction in IL-6, IL-1β, TNF-α, and iNOS	Hu et al. [115]
	Polysaccharides	RAW 264.7 macrophages	Modulation of MAPK and PPAR signaling pathway to downregulate inflammatory cytokines	Hu et al. [115]
	Mycelium extract	Monosodium iodoacetate(MIA)-induced osteoarthritis in Sprague Dawley rats	Suppression of inflammatory factors and elements associated with cartilage matrix degradation	Shin et al. [240]
	Ethanol extract	Dextran sulfate sodium (DSS) colitis-induced mice	Ameliorate pathological characteristics of colitis and reduce NF-κB, iNOS, Cox-2, and MAPK	Song and Park [241]
	Ethanol extract	LPS-stimulated RAW264.7 cells	Reduce levels and expressions of NO, PGE_2_, iNOS, NF-κB, Cox-2, and TNF-α	Song and Park [241]
	*P. linteus* grown on Panax ginseng media	RBL-2H3 cells	Suppress phosphorylation of spleen associated tyrosine kinase, GRB2-associated-binding protein 2 (Gab2), and extracellular signal-regulated kinases proteins	Park [242]
	*P. linteus* grown on Panax ginseng media;	LPS-stimulated RAW264.7 cells	Inhibit NO, IL-6, and TNF-α production	Park [242]
	*P. linteus*-fermented broth	LPS-stimulated RAW264.7 cells and murine primary peritoneal exudate macrophages	Inhibit NO, NF-κB, and TNF-α expressions	Lin et al. [145]
	Inotilone	LPS-stimulated RAW264.7 cells	Inhibit NO production, iNOS, NF-κB, and MMP-9 expressions, and LPS-induced ERK, JNK, and p38 phosphorylation	Huang et al. [212, 243]
Antiviral	Methanol extract	Newcastle virus (NDV)-infected baby hamster kidney cells	Suppressed NDV-induced syncytium formation at 12.5 µg/mL	Lee [244]
	Inotilone and 4-(3,4-dihydroxyphenyl)-3-buten-2-one from fermentation broth	H1N1 neuraminidase inhibition assay	10 uM of both inhibited the formation of visible cytopathic effects, with IC50 values lower than Oseltamivir	Hwang et al. [13]
	Hispidin and hypholomine B from mycelial ethanol	Severe acute respiratory syndrome coronavirus 2 (SARS-CoV-2) virus-infected HepG2 cells	Reduced the expression of angiotensin-converting enzyme 2 (ACE2) gene to minimize viral entry Blockage of spike receptor-binding domain Inhibition of 3CL protease by hypholomine B	Li et al. [35, 38]

Park et al. [227] reported that the ethyl acetate extract of *T. linteus* grown in germinated brown rice induced apoptosis in HT-29 cells via the induction of cell cycle arrest at the G0/G1 phase and DNA fragmentation. HT-29 cells were also susceptible to the n-hexane layer of ethyl acetate extract from *T. linteus* which showcased potent anticancer effects with an IC_50_ of 69.8 µg/mL at the 48 h time point [228]. Moreover, the methanol extract of *T. linteus* also displayed anticancer effects in SW-480 and HCT-116 cells where an increase in anti-migratory marker E-cadherin and a decrease in pro-migratory/pro-invasive proteins N-cadherin and Vimentin were observed with higher selectivity towards SW-480 cells [229]. Besides, extracts of *T. linteus* grown on germinated brown rice could increase the sensitivity of KRAS-mutated SW480 colon cancer cells to cetuximab with a reduced number of colonies in clonogenic assay and increased apoptotic rate [230]. Recent research has also revealed that *T. linteus* possesses potent anti-breast cancer proliferative activity by downregulating marker of proliferation Kiel 67 (MKI67), Human epidermal growth factor receptor 2 (HER2)*,* epidermal growth factor receptor (EGFR)*, *murine double minute 2 (MDM2)*, *TNFα, and phosphatidylinositol-4,5-bisphosphate 3-kinase catalytic subunit alpha (PI3KCA) expression [245].

A novel polysaccharide isolated from *T. linteus* exhibited stronger inhibitory effects than 5-fluorouracil on murine sarcoma cancer cells (S-180) at 12 and 24 h, with inhibition rates of up to 93% at 72 h at a concentration of 200 µg/mL [126]. The polysaccharide’s potent in vitro anticancer properties were found to be mediated by a decreased expression of Bcl-2 with a rise in pro-apoptotic Bax protein expression, indicating that the observed anticancer effect was exerted via stimulation of apoptosis. Furthermore, two bioactive compounds (PL-ES and PL-I-ES) isolated from *T. linteus* were also found to exert apoptosis-associated anticancer effects on a total of 10 different cancer cell lines where 100 g/mL of PL-ES could suppress growth in all 10 cancer cells, while 100 and 250 g/mL of PL1 was able to cause significant growth reduction in four and seven cancer cells types respectively [231].

Besides from the above mentioned constituents, hispolon from *T. linteus* also exhibited anticancer effects in MCF-7, MDA-MB-231, T24, and J82 cells where it suppressed p53 mutant (MDA-MB-231, T24, and J82) and wild-type (MCF7) cells regardless of p53 status [232]. The extract induced apoptosis in the mentioned cancer cells, which was found to be mediated via chromatin condensation and an increase in poly (ADP-ribose) polymerase (PARP). Additionally, Liu et al. [233] reported that Atractylenolide I isolated from *T. linteus* was effective in gastric cancer cachexia management, where it was found to be significantly more effective than fish-oil-enriched nutritional supplementation at improving appetite, Karnofsky performance status, and decreasing proteolysis-inducing factor positive rates, suggesting the use of *T. linteus* as not only an anticancer agent, but also as a functional food during cancer treatment (Fig. 5).

#### Hypoglycemic and hypolipidemic

The hypoglycemic properties of *T. linteus* extract from mycelia of solid-state culture was observed in diabetic rat models by Liu et al. [10], as evidenced by decreased serum glucose, glycated serum proteins, and insulin levels along with increased expressions of glucokinase and GLUT2 8 weeks after treatment with 120 and 600 mg/kg. A similar study by Kim et al. [234] also found that exo-polymers produced from the submerged cultures of *T. linteus* were also able to induce hypoglycemic effects on streptozotocin-induced diabetic rats, which was proposed to be through reparation of streptozotocin-exposed pancreatic β-cells, thus increasing insulin secretion leading to effective clearance of blood glucose. This finding is also supported by Sonawane et al. [246], where the crude polysaccharide of another mushroom from the *Phellinus* family, named *Phellinus badius* was also expected to exert the same effects on streptozotocin-induced diabetic rats. Moreover, polysaccharides isolated from *T. linteus* were found to improve insulin resistance via amplification of the population of short-chain fatty acids (SCFAs)-producing bacteria in the gut of streptozotocin induced-type 2 diabetes mellitus mice, which resulted in increased levels of SCFA that sustained the intestinal barrier function and thus caused reduction in LPS content in blood [125]. Consistent finding was also reported by Zhao et al. [124] in alloxan-induced diabetic mice, where mean percentage decrease in blood glucose levels of 22.35%, 16.35%, and 15.19% at concentrations of 100, 200, and 400 mg/kg of *I. obliquus* polysaccharides respectively was recorded.

Besides from its hypoglycemic properties, *T. linteus* extract from mycelia of solid-state culture is also capable of exerting hypolipidemic effects in HFD-induced rat models, with significant reductions in the levels of TG, TC, free fatty acids, and LDL-C in groups treated with 120 mg/kg and 600 mg/kg of the extract [10]. The extract’s hypolipidemic activities are proposed to be mediated through regulation of the expression of genes involved in lipid transport and metabolism, including an increase levels of fatty acid β-oxidation-associated genes acyl-CoA oxidase 1 (ACOX1) and CPT1A, elevation of amount of LDL receptors, and reduction of rate-limiting enzyme 3-hydroxy-3-methylglutaryl-CoA reductase (HMGCR), which together contribute to its potent hypolipidemic effects [125]. Similarly, administration of ethyl acetate fraction from *T. linteus* mycelia (PL-EA) for four weeks was able to improve body weight, hepatic lipid accumulation, and fasting blood glucose levels in HFD-induced mice through upregulating peroxisome proliferator-activated receptor gamma coactivator 1-alpha (PGC-1α) and adiponectin while downregulating glucokinase and SREBP-1c [138]. Furthermore, the major purified compounds of hispidin and hypholomine B in PL-EA significantly reduced the level of oleic and palmitic acids-induced lipid accumulation in HepG2 cells to contribute to the alleviation of non-alcoholic fatty liver in treated groups.

#### Antioxidant

According to Lee et al. [235], hispidin isolated from *T. linteus* was found to protect MIN6N β-cells of the pancreas from oxidative stress, where treatment with hispidin between the range of 10–70 µM revealed notable ROS scavenging activity in a dose-dependent manner, with the highest concentration having an activity of about 65%. The antioxidant properties of the extract were found to be mediated by an inhibition of caspase-3 activity, thus preventing the initiation of oxidative-stress induced apoptosis [235]. In addition, three constituents obtained from the culture broth of *T. linteus*, namely caffeic acid, inotilone, and 4-(3,4-Dihydroxyphenyl)-3-buten-2-one, were found to possess potent antioxidant activity in DPPH, ABTS and FRAP assay, while (2E,4E)-γ-Ionylideneacetic acid, Phellilane H, and (2E,4E)-4′-Hydroxy-γ-ionylideneacetic acid showed weak reducing power [133]. It should also be noted that caffeic acid, inotilone, 4-(3,4-Dihydroxyphenyl)-3-buten-2-one recorded higher antioxidant activity than butylated hydroxyanisole and Trolox in FRAP assay.

Three polysaccharides isolated from *T. linteus* exerted strong DPPH radical scavenging activity of between 58 to 72% at a concentration of 2 mg/mL [119]. Another study reported that hot water polysaccharide extracts of *T. linteus* fruiting bodies demonstrated notable antioxidant capabilities, with DPPH scavenging activities stronger than that of ascorbic acid at 5 mg/mL and above [236]. In addition, the stronger antioxidant activity of *T. linteus* was demonstrated by a significantly lower EC_50_ value than that of *Aspergillus brasiliensis* and *Agaricus bisporus*, further highlighting the potent antioxidant effects of *T. linteus* [236].

#### Hepatoprotective

Wang et al. [237] reported that the polysaccharides of *T. linteus* exhibited protective effects on thioacetamide liver fibrosis-induced mice via the regulation of the heat shock, oxidative stress, amino acid and nucleic acid metabolic pathways. Extracts isolated from *T. linteus* grown on germinated rice were also found to be capable of reducing peroxidation products in the liver of carbon tetrachloride-induced mice and decreasing cytochrome P450 2E1 (CYP2E1) protein expression [238]. Moreover, a decrease in glutamic transaminase enzyme released was also observed in H_2_O_2_-injured rat hepatocytes treated with 100 µg/ml methanol extract of *T. linteus* mycelial culture [239]. Further investigations revealed that the ethyl acetate fraction from the methanol extract of mycelial culture displayed 68.9 ± 5.3% protection to H_2_O_2_-injured hepatocytes and 46.8 ± 3.9% protection to galactosamine-injured hepatocytes, demonstrating the potent hepatoprotective effects of *T. linteus*.

#### Anti-inflammatory

*T. linteus* polysaccharides have been shown to substantially reduce the expression of key inflammatory cytokines, IL-6, IL-1β, and TNF-α, both in vivo in murine models induced with DSS and in vitro in LPS-stimulated RAW 264.7 macrophages through modulation of the MAPK and PPAR signaling pathways [115]. Similar conclusions were made by Shin et al. [240], who demonstrated the ability of *T. linteus* to deactivate NF-κB signaling through the inhibition of p38 MAPK, subsequently suppressing both inflammatory factors and elements associated with cartilage matrix degradation, thereby exerting a potent chondroprotective effect through simultaneous mitigation of ROS production and inflammation [240].

Extracts from *T. linteus* growth on germinated brown rice could inhibit NO and PGE2 production as well as reduce iNOS, NF-κB, COX-2, and TNF-α expressions to ameliorate pathological changes of colitis [241], while *T. linteus* grown on Panax ginseng media could suppress phosphorylation of spleen associated tyrosine kinase, GRB2 associated binding protein 2 (GAB2), and extracellular signal-regulated kinases required for degranulation and release of inflammatory cytokines [242]. Similar findings were obtained in a study by Lin et al. [145], where *T. linteus*-fermented broth exhibited dose-dependent inhibition of NO, NF-κB, and TNF-α in RAW264.7 cells and murine primary peritoneal exudate macrophages. A notable observation is the presence of high amounts of hispolon in the sample that showed the strongest anti-inflammatory activities, suggesting the important role of this compound in the inflammation-reducing functions of *T. linteus* [145]. Another compound isolated from *T. linteus*, inotilone, has been demonstrated to possess potent anti-inflammatory properties, capable of inhibiting NO production, protein expression of iNOS, NF-κB, and MMP-9, and suppression of LPS-induced ERK, c-Jun N-terminal kinase (JNK), and p38 phosphorylation in RAW264.7 macrophages [243]. Taken together, the abovementioned studies highlight the potent anti-inflammatory abilities of *T. linteus* and its potential for therapeutic applications.

#### Antiviral

Inhibition studies revealed a dose-dependent inhibitory activity of *T. linteus* methanol extract on α-glucosidase, a crucial component for the morphogenesis of enveloped viruses [244]. Due to the similarity between syncytium formation of Newcastle disease virus (NDV) and HIV, the inhibition effect of the extract on hemagglutinin-neuraminidase protein of NDV expressed on baby hamster kidney cells were measured, leading to the finding that *T. linteus* methanol extract was capable of suppressing NDV-induced syncytium formation at 12.5 µg/mL, similar to that of α-glucosidase inhibition [244]. This suggests the potential use of *T. linteus* against viruses such as HIV, HBV, and dengue virus, which are known to be highly sensitive to glucosidase inhibition.

Besides glucosidase, two constituents of *T. linteus* fermentation broth, inotilone as well as 4-(3,4-dihydroxyphenyl)-3-buten-2-one demonstrated strong neuraminidase inhibitory effects [13]. Microscopic findings revealed that 10 µM of both isolated constituents inhibited the formation of visible cytopathic effects, with IC_50_ values lower than that of Oseltamivir (61 and 52 µM respectively compared to 64 µM), suggesting the potential of *T. linteus*-derived constituents as stronger neuraminidase inhibitors of Influenza A H1N1 virus than the commonly used Oseltamivir treatment [13]. In addition, recent findings by Li et al. [35, 38] reported that hispidin and hypholomine B from the mycelial ethanol extract of *T. linteus* reduced the expression of angiotensin-converting enzyme 2 (ACE2) gene in HepG2 cells and blocked the spike receptor-binding domain to minimize the entry of the severe acute respiratory syndrome coronavirus 2 (SARS-CoV-2) virus, further demonstrating the strong antiviral and therapeutic potential of *T. linteus* for viral infections.

## Medical evidence through clinical trials

### Taiwanofungus camphoratus

The promising health-promoting and therapeutic effects of *T. camphoratus* have been demonstrated through both in vitro and in vivo experiments, as discussed previously. In recent years, clinical trials have been conducted to further validate the pharmacological activities of the medicinal mushroom in the biological conditions of the human body (Table 5).

**Table 5:** Clinical trials carried out on *Taiwanofungus camphoratus*, *Inonotus obliquus*, and *Tropicoporus linteus*

Intervention	Dosage form	Subject recruitment	Clinical Outcome	Reference
*T. camphorata*	420 mg three capsules per day	41 patients have systolic blood pressure between 130 and 179 mmHg or diastolic blood pressure between 85 and 109 mmHg	Decrease in both systolic and diastolic blood pressure among individuals in the TC mycelium group and risk of cardiovascular disease, likely influenced by the inhibition of renin secretion and modulation of the renin-angiotensin system	Chen et al. [58, 247]
Golden-*T. camphorata*	300 mg tablets daily	80 participants with elevated γ-glutamyl transferase levels	Decline in serum ALT and AST levels, as well as reduced levels of triglycerides in patients, exhibited hepatoprotective effects	Yen et al. [248]
*T. camphorata*	Routine chemotherapy regimens with 20 mL, twice a day	37 advanced cancer patients	No significant benefit on fatigue or quality of life related outcomes	Tsai et al. [249]
*I. obliquus*	1 tablespoon, 3 times/day, 20–30 min before meals	50 patients with psoriasis	92% reduction of Complete psoriatic eruptions. Gastrointestinal tract symptoms were modified or disappeared completely	Dosychev and Bystrova [250]
*I. obliquus*	4.5 ml/day, 45 ml/day, 90 ml/day in each respective groups	58 patients with peptic ulcers	Temporary reduction in pain, and higher doses results in a greater decrease in pain	Fedotov and Yu [251]
*T. linteus*	1,000 mg/day and 1,500 mg/day daily in each respective groups	30 participants with a history of upper respiratory infections	Has immune-enhancing potential, and showed anti-inflammatory properties	Ku and Kang [252]
*T. linteus*	1,000 mg/day and 2000 mg/day daily in each respective groups	24 patients having knee osteoarthritis for 6 months or long	Relieves pain from knee arthritis and reduce markers of inflammation	Ryu, Lee, and Kang [253]
*T. linteus*	1100 mg 3 times per day orally	323 pancreatectomy patients	Enhances adherence to chemotherapy after curative resection, reduces chemotherapy-related toxicities, and improves survival rates	Sung Hwan Lee et al. [254]

Chen et al. [247] found that 8 weeks of oral *T. camphoratus* mycelium treatment could induce significant reductions in systolic and diastolic blood pressure, which was proposed to be through interaction with the renin-angiotensin system to lower blood pressure by inhibiting renin secretion. The historical use of *T. camphoratus* as a traditional Chinese medicine for treating hypertension further substantiates these contemporary findings [255]. Golden-*T. camphoratus* administration was also capable of reducing AST, ALT, and TG levels with no significant effects on general safety parameters, suggesting the use of Golden-*T. camphoratus* as a safe and effective hepatoprotective agent [248].

In addition, a study conducted by Tsai et al. [249] to explore the combined effects of *T. camphoratus* treatment and chemotherapy found that although significant enhancements in fatigue or quality of life were not observed, *T. camphoratus* did contribute to improved sleep quality. Nevertheless, the potential benefits were clouded by adverse effects, including gastrointestinal discomfort and a reduction in platelet counts within a month of treatment, where the precise relationship between these effects and *T. camphoratus* remains to be fully elucidated. Although prior in vitro and in vivo studies such as that conducted by Huang et al. [256] have highlighted the capability of *T. camphoratus* to enhance the sensitivity of colon cancer cells to chemotherapy, these promising results did not translate directly to clinical trials, as evidenced by the divergent outcomes observed by Tsai et al. [249] due to potentially different interactions with the complexities of human clinical contexts. This underlines the need for cautious interpretation and further rigorous investigation to ascertain these findings in clinical applications.

### Inonotus obliquus

Numerous in vitro and in vivo studies have highlighted the potential health benefits and therapeutic effects of *I. obliquus*, thus prompting the initiation of several clinical trials to explore the pharmacological activities of *I. obliquus* in human subjects.

In a study undertaken by Dosychev and Bystrova [250], it was found that 38 patients experienced a complete disappearance of psoriatic eruptions, while 8 patients exhibited significant improvement in related symptoms. This outcome resonates with a related study that highlighted the immune-promoting effect of *I. obliquus*, indicating its suitability in the context of molecular mechanisms of action in immunological disease driven by TNF-α, which pertains to conditions such as psoriasis [257]. Additionally, noticeable relief or complete resolution of gastrointestinal tract symptoms was observed [250], which agrees with an in vivo investigation conducted by Hu et al. [258] that reported that *I. obliquus* polysaccharide contributes to shaping the gut microbiota composition toward a healthier profile at the genus level.

An investigation carried out by Fedotov and Yu [251] found that *I. obliquus* was capable of reducing pain related to peptic ulcers, with more substantial effects seen at higher doses. However, interestingly, the cessation of *I. obliquus* treatment resulted in the return of pain levels to their original state. These findings are aligned with a related study conducted on rats which demonstrated the effective antiulcer activity of *I. obliquus,* further validating the therapeutic effect of this medicinal mushroom [259].

Despite its widespread global utilisation and the multitude of experiments conducted, the available corpus of peer-reviewed literature encompasses solely two clinical studies, both conducted over four decades ago, as previously indicated [250, 251]. It is important to underscore that no controlled trials assessing the safety of *I. obliquus* were identified, and no pertinent studies were found to be registered on Clinicaltrials.gov. Analogous to many mushroom supplements, the prevailing evidence concerning safety and efficacy heavily relies on the extensive historical use of *I. obliquus*. In light of this, further comprehensive studies, particularly through rigorous clinical trials, are imperative to establish a more nuanced and comprehensive understanding of *I. obliquus*'s impact on the human body [260].

### Tropicoporus linteus

A recent randomized, double-blind, placebo-controlled pilot trial conducted on participants with a history of upper respiratory infections validated the immunomodulatory effect of *T. linteus* as evidenced by heightened natural killer cell activity and elevated IL-6, immunoglobulin G1 (IgG1), IgG2, and IgM levels [252]. Another pilot clinical study revealed the ability of *T. linteus* to relieve pain resulting from knee osteoarthritis and return markers of inflammation to normal values, suggesting the potential of *T. linteus* in alleviating knee arthritis symptoms [253]. This is in line with the observation made by Kim et al. [261] where *T. linteus* was able to suppress collagen-induced arthritis in vivo by reducing anti-type II collagen IgG and IgG2a antibodies as well as various cytokines including IL-12, TNF-α, and IFN-γ in mice, further highlighting the pivotal role of *T. linteus* in preventing and treating joint inflammation.

In a study conducted by Sung et al. [254], patients with pancreatic cancer who underwent pancreatectomy were recruited over the course of 19 years and retrospectively analysed to examine the impact of *T. linteus* on adherence to postoperative adjuvant treatment. Remarkably, *T. linteus* medication increased patient adherence to postoperative chemotherapy to contribute to overall long-term oncologic outcomes, further highlighting the potential application of *T. linteus* as not only an anticancer agent as evidenced by in vitro and in vivo studies, but also as an adjuvant to improve outcomes to conventional chemotherapy.

## Conclusion

This review has extensively examined the diverse biological activities, therapeutic effects, and pharmacological impacts of three notable mushrooms: *T. camphoratus*, *I. obliquus*, and *T. linteus*. Throughout numerous in vitro and in vivo studies, these mushrooms have revealed their potential through bioactive compounds that hold promise for human well-being. The growing interest in natural products as alternative treatments for conditions such as cancer and viral infections is evident from the increased scholarly and societal attention. Nonetheless, it is imperative to underscore the need for further comprehensive clinical trials that focus on safety, toxicity, and potential risks. Such investigations would offer robust scientific foundations and practical insights for optimising the biological effectiveness of these mushrooms while minimising any adverse effects. This will also encourage the development of mushroom products in not only medicinal applications, but also its exploitation in the cosmetic industry due to their excellent antioxidant, anti-aging, and moisturizing properties, ensuring a wider accessibility to the manifold benefits these mushroom species offer to a broader population.

## Data Availability

Not applicable.

## Abbreviations

ACC: Acetyl-CoA carboxylase
ACE: Angiotensin-converting enzyme
ACOX: Acyl-CoA oxidase
ADP: Adenosine diphosphate
ALT: Alanine aminotransferase
AMPK: Adenosine monophosphate-activated protein kinase
AST: Aspartate transaminase
CDK: Cyclin-dependent kinase
COX: Cyclooxygenase
CPT-1A: Carnitine palmitoyltransferase 1A
CYP2E1: Cytochrome P450 2E1
DSS: Dextran sulfate sodium
ECG: Epicatechin-3-gallate
EGCG: Epigallocatechin-3-gallate
EGFR: Epidermal growth factor receptor
EMT: Epithelial-mesenchymal transition
ERK: Extracellular signal-related kinases
FCV: Feline calicivirus
FHV: Feline herpesvirus 1
FIPV: Feline infectious peritonitis virus
FIV: Feline influenza virus
FPV: Feline panleukopenia virus
FXR: Farnesoid X receptor
GLUT4: Glucose transporter 4
GSH: Glutathione
HBeAg: Hepatitis B e-antigen
HBV: Hepatitis B virus
HCV: Hepatitis C virus
HDL: High-density lipoprotein
HDL-C: High-density lipoprotein cholesterol
HER: Human epidermal growth factor receptor
HFD: High-fat diet
HIV: Human immunodeficiency virus
HPLC: High-performance liquid chromatography
HSV: Herpes simplex virus
IBA-1: Ionized calcium-binding adapter molecule-1
IFN: Interferon
IL: Interleukin
iNOS: Inducible-nitric oxide synthase
IOPS: *I. obliquus* Polysaccharides
JNK: C-Jun N-terminal kinase
LDL: Low-density lipoprotein
LDL-C: Low-density lipoprotein cholesterol
LPS: Lipopolysaccharide
MAPK: Mitogen-activated protein kinases
MDA: Malondialdehyde
MDM: Murine double minute 2
MDR: Multidrug resistance
MMP: Matrix metalloproteinase
NDV: Newcastle disease virus
NF-κB: Nuclear factor kappa B
NO: Nitric oxide
Nrf: Nuclear factor erythroid 2–related factor 2
p-Akt: Phospho-protein kinase B
PARP: Poly (ADP-ribose) polymerase
PGC-1α: Peroxisome proliferator-activated receptor gamma coactivator 1-alpha
PGE2: Prostaglandin E2
PI3K: Phosphoinositide 3-kinase
PI3KCA: Phosphatidylinositol-4,5-bisphosphate 3-kinase catalytic subunit alpha
PPAR: Peroxisome proliferator-activated receptor
Rb: Retinoblastoma tumour suppressor protein
ROS: Reactive oxygen species
SCFA: Short-chain fatty acid
SFC: Supercritical fluid chromatography
SHP: Small heterodimer partner
SOD: Superoxide dismutase
SREBP-1C: Sterol-regulatory element binding protein-1C
STAT3: Signal transducer and activator of transcription 3
TC: Total cholesterol
TG: Triacylglycerol
TNF: Tumour necrosis factor
UV: Ultraviolet
